# The Chloroplast Ribonucleoprotein CP33B Quantitatively Binds the *psbA* mRNA

**DOI:** 10.3390/plants9030367

**Published:** 2020-03-17

**Authors:** Marlene Teubner, Benjamin Lenzen, Lucas Bernal Espenberger, Janina Fuss, Jörg Nickelsen, Kirsten Krause, Hannes Ruwe, Christian Schmitz-Linneweber

**Affiliations:** 1 Institute of Biology, Department of Life Sciences, Humboldt University Berlin, 10115 Berlin, Germany; teubner.marlene@gmx.de (M.T.); benjamin.lenzen@outlook.com (B.L.); lucas.bernal.espenberger@gmail.com (L.B.E.); hannes.ruwe@hu-berlin.de (H.R.); 2 Department of Arctic and Marine Biology, UiT The Arctic University of Norway, Framstredet 39, 9019 Tromsø, Norway; j.fuss@ikmb.uni-kiel.de (J.F.); kirsten.krause@uit.no (K.K.); 3 Department Biologie I, Botanik, Ludwig-Maximilians-Universität, 82152 Planegg-Martinsried, Germany; joerg.nickelsen@lrz.uni-muenchen.de

**Keywords:** RNA processing, RNA-binding, *psbA*, RNA-Bind-n-Seq, organelle, *Arabidopsis thaliana*, D1, Photosystem II, RNA recognition motif

## Abstract

Chloroplast RNAs are stabilized and processed by a multitude of nuclear-encoded RNA-binding proteins, often in response to external stimuli like light and temperature. A particularly interesting RNA-based regulation occurs with the *psbA* mRNA, which shows light-dependent translation. Recently, the chloroplast ribonucleoprotein CP33B was identified as a ligand of the *psbA* mRNA. We here characterized the interaction of CP33B with chloroplast RNAs in greater detail using a combination of RIP-chip, quantitative dot-blot, and RNA-Bind-n-Seq experiments. We demonstrate that CP33B prefers *psbA* over all other chloroplast RNAs and associates with the vast majority of the *psbA* transcript pool. The RNA sequence target motif, determined in vitro, does not fully explain CP33B’s preference for *psbA*, suggesting that there are other determinants of specificity in vivo.

## 1. Introduction

The maturation and translation of chloroplast RNAs depends on numerous RNA-binding proteins (RBPs). With few exceptions, all RBPs involved in chloroplast RNA metabolism are encoded in the nucleus and are post-translationally imported into plastids. The largest family of RBPs in chloroplasts are the pentatricopeptide repeat proteins (PPR proteins), which interact specifically with one or few chloroplast transcripts [1]. In addition to PPR proteins, several smaller RBP families exist in the chloroplast, including the family of chloroplast ribonucleoproteins (cpRNPs), whose members are extremely abundant, bind multiple mRNAs, and which are regulated in response to various biotic and abiotic signals [2].

cpRNPs are characterized by their conserved domain structure. An N-terminal chloroplast import signal (transit peptide), which is cleaved off after transport into the chloroplast, is followed by an acidic domain and two RNA recognition motif (RRM) domains. cpRNPs are able to interact with different nucleic acids (ssDNA, dsDNA and RNA) [3,4], but the strongest association in vitro is with ribonucleic acids [5]. In vitro and in vivo interactions with specific plastid mRNAs were demonstrated [6], cumulating in transcriptome-wide binding studies that showed a broad range of target mRNAs for various cpRNPs [7,8,9]. rRNAs and intron-less tRNAs are not or only weakly bound [6,7,10]. Since the cpRNPs do not co-fractionate with polysomal RNAs [6,8,11], they are mainly attributed a function prior to translation within posttranscriptional processes.

Prediction algorithms for subcellular localization and shotgun proteome analysis identified all ten cpRNPs of Arabidopsis in the chloroplast [summarized in 2]. Fluorescence microscopy of GFP fusion proteins confirmed the chloroplast localization [8,12,13,14]. Within the chloroplasts, the stroma is the main destination of cpRNPs, with small amounts also being associated with thylakoids. This was proven by immunological analyses for the five cpRNPs from tobacco [6].

The expression of cpRNPs is regulated by various external and internal signals. Light especially leads to an accumulation of cpRNPs [summarized in 2]. In general, cpRNPs are involved in a variety of posttranscriptional processes, including 3’-end processing of RNAs [15], RNA editing [16,17], RNA splicing [7], and RNA stabilization [7,8,10]. Some of these processes are modulated by cpRNPs in response to environmental cues and several cpRNPs have been implicated in different acclimation and stress responses [2,7,13,18]. Such a multi-level and far-reaching regulation by multiple external and internal stimuli is unknown for most other chloroplast RBPs, including PPR proteins. cpRNPs are thus considered as prime candidates for post-transcriptional regulators of plastid gene expression [19].

A particularly interesting case of chloroplast gene regulation is the light-induced translation of *psbA*, which codes for the D1 protein, the core subunit of photosytem II [20,21,22,23]. D1 is constantly damaged, most pronouncedly by excess light and other unfavourable conditions, i.e., cold. As a consequence, D1 is constantly synthesized for the repair of PSII [24,25,26]. Moreover, regulated D1 synthesis for de novo biogenesis of PSII during cell growth requires additional regulatory levels of *psbA* mRNA translation. Consistently, a number of proteins have been co-purified with the *psbA* mRNA in Chlamydomonas, spinach, Arabidopsis and maize, and or have been identified by genetic analyses [27,28,29,30,31,32,33,34,35]. Among the proteins co-precipitating with the *psbA* mRNA was CP33B (AT2G35410) [9]. In contrast to other cpRNPs, CP33B appears to have a clear preference for the *psbA* mRNA and does show comparatively little binding to other mRNAs [9]. We here analyzed the binding preference of CP33B in more detail and identified a main target sequence motif in vitro.

## 2. Results

### 2.1. CP33B Localizes to the Chloroplast

**Figure 1:**
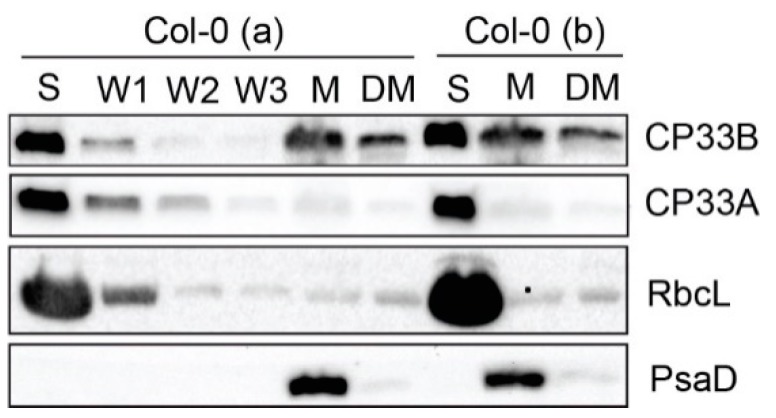
CP33B localizes to stroma and membrane fraction of chloroplasts. Chloroplasts of two wild type replicates (Col-0 a and b) were separated into stroma (S) and membranes (M). Membranes were washed 5 times. A membrane aliquot was treated with 0.5% sodium deoxycholate and the supernatant was harvested after treatment (DM). From each fraction, including the first three washing steps (W1, W2, W3) of Col-0 (a), equal volume fractions were separated electrophoretically by SDS PAGE and transferred to a nitrocellulose membrane. Besides the immunological detection of CP33A (antibody peptide a) and CP33B, antibodies for RbcL (large subunit of RuBisCO (ribulose 1.5 bisphosphate carboxylase/oxygenase)) and PsaD (subunit of photosystem I) served as stroma and membrane markers, respectively. The controls (RbcL, CP33A, PsaD) are reprinted with permission from Wiley; original Figure 2c in [8]; © 2020 John Wiley & Sons Ltd, Hoboken, New Jersey, USA.

Arabidopsis CP33B is predicted to be a chloroplast protein based on algorithms such as Predotar and TargetP [2]. This is supported by the recent finding that a maize orthologue of CP33B was isolated from chloroplast stroma by precipitating the *psbA* mRNA [9]. We analyzed the location of Arabidopsis CP33B experimentally by assaying cell fractions immunologically. Chloroplasts were isolated from 14-day-old wild type plants and separated into membranes and soluble proteins (= stroma). The membranes were washed several times and a part was solubilized with the anionic detergent sodium deoxycholate (0.5%) (Figure 1). From each fraction, equal volume portions were separated by SDS PAGE. A PsaD antibody, which detects a peripheral subunit of photosystem I, was used as a membrane marker. No signal is detected for PsaD in the stroma fractions, a strong signal in the membranes and only a very weak signal in the detergent-treated membranes. Thus, not even peripheral membrane proteins such as PsaD are released from the membrane by the treatment. The detection of RbcL, the large subunit of the ribulose 1,5 bisphosphate carboxylase/oxygenase (RuBisCO) located in the stroma, serves as a marker for the stroma fraction. The Western analyses showed that the majority of CP33B, like CP33A, is found in the stroma. In contrast to CP33A, which showed only a very weak membrane association, CP33B was also present to a considerable degree in the membrane fraction. (Figure 1). We quantified the CP33B signals and found that about 75% of CP33B is found in the stroma and about a quarter in the membrane fraction. Since CP33B can be easily dissolved from the membrane fractions with the mild detergent sodium deoxycholate, the membrane-bound part of CP33B is not integrated into the membranes, but is likely to be bound peripherally, via weak interactions to the membranes. Overall, the largest proportion of CP33B localizes in the stroma, in line with all previous analyses of cpRNP proteins.

**Figure 2:**
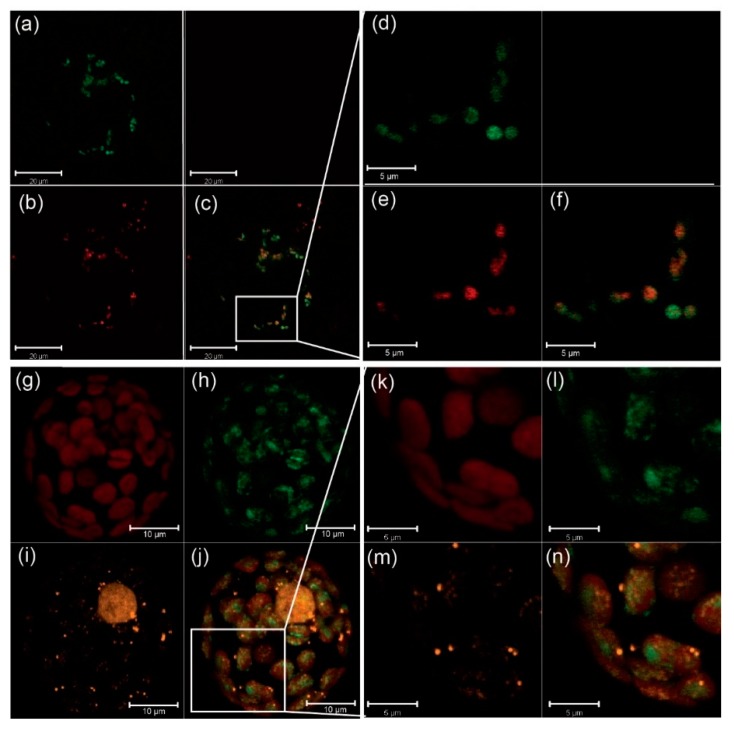
CP33B is not associated with nucleoids. (**a–f**) Images of CP33B:GFP-transformed Arabidopsis protoplasts. (**d**), (**e**), and (**f**) are enlargements of the white box area of (**c**) respectively. GFP fluorescence (**a**,**d**, green); chlorophyll autofluorescence (b and e, red); superimposition of images (**a**) and (**b**) in (**c**), and (**d**) and (**e**) (**f**), respectively. (**g–n**) Images of co-transfection experiments of CP33B:GFP and PEND:dsRed. (**k**–**n**) are enlargements of the white box area of (**g–j**). Chlorophyll autofluorescence (**g**,**k**, red); GFP fluorescence (**h**,**l**, green); dsRed fluorescence (**i**,**m**, orange); superimposition of images (**g**–**i**) (in **j**) and (**k**–**m**) (in **n**), respectively. The white bars represent 20, 10 and 5 μm, as indicated.

The stroma localization of CP33B was further analyzed by fluorescence microscopy. For this, the coding sequence of CP33B was fused with a GFP tag and transiently expressed in protoplasts. The GFP fluorescence signal is mainly diffusely distributed in chloroplasts, with a partial overlap with chlorophyll autofluorescence (Figure 2a–f). RBPs have been found to co-fractionate with nucleoid preparations [36], and thus we decided to test for nucleoid association as well. To analyze a potential co-localization of CP33B with nucleoids, a co-transfection was performed with a vector expressing PEND:dsRed. PEND binds to plastid DNA and can therefore be used as a marker for nucleoids [37]. The fluorescence images show little or no overlap of CP33B GFP and the PEND dsRed signal (Figure 2g–n), which makes an association of CP33B with nucleoids unlikely. In sum, CP33B localizes predominantly to the chloroplast stroma with a minor fraction of CP33B attached to membranes.

### 2.2. CP33B Has a Preference for the psbA mRNA Over Other Chloroplast Transcripts

**Figure 3:**
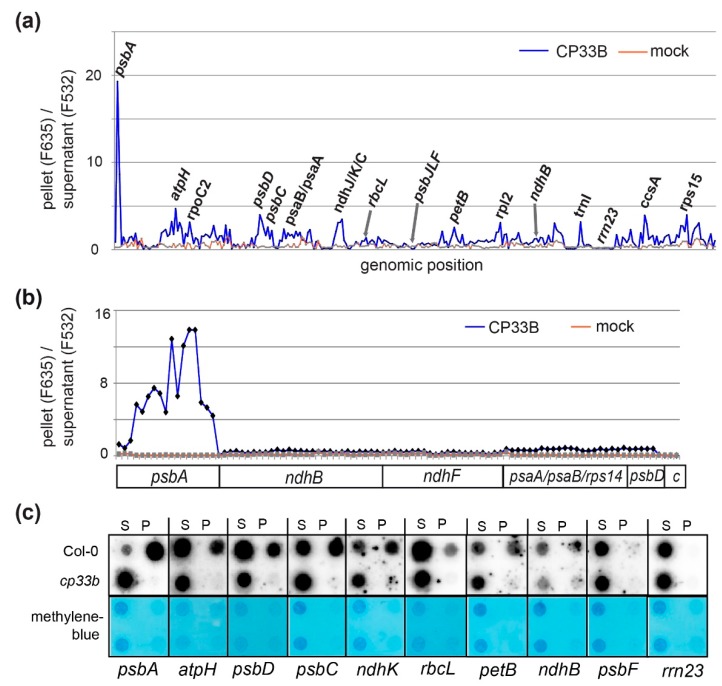
CP33B associated with multiple plastid transcripts, but foremost with the *psbA* mRNA. (**a**) RIP-chip analysis of RNA co-precipitated with CP33B using a microarray consisting of PCR products representing the entire Arabidopsis chloroplast genome with, on average, 1 kb fragments. Ratios of fluorescence signals from co-precipitated RNA (F635) and unbound RNA (F532) were plotted against the position on the chloroplast genome. Three biological replicates from CP33B immunoprecipitations and as control, two replicates of precipitations with the corresponding pre-immune serum and an IP on stroma of *cp33b* null mutants were performed. The precipitate/supernatant ratios (median (median of ratios)) were normalized to the sum of the median values of the ribosomal RNAs of the supernatants (median F532; Table S1). Only selected peaks are labelled. (**b**) Oligonucleotide RIP-chip analysis for the interaction of CP33B with a selection of chloroplast transcripts. CP33B immunoprecipitations were performed from wild-type stroma with anti CP33B (four biological replicates) and as a control with the corresponding pre-immune serum (three biological replicates). Shown are the median of ratios of fluorescence signals from co-precipitated RNA (F635) and unbound RNA (F532; sequences of oligonucleotides and data in Table S2). Each plastid transcript, except for negative controls (**c**), is represented by several oligonucleotides (50 nt long; for a zoom-in see Figure S1). (**c**) The CP33B immunoprecipitations were performed on wild type stroma and *cp33b* null mutants. Equal volumes of isolated RNA from precipitate (P) and supernatant fraction (S) were transferred onto nylon membranes and hybridized with different radiolabeled RNA probes. The methylene blue staining of the nylon membranes reflects the total RNA content of each fraction. The analysis was repeated twice for *psbA* and once more for *rbcL* and rrn23, with similar results.

Previously, we had shown by RNA-co-immunoprecipitation and next-generation sequencing (RIP-Seq) that CP33B associates with a number of chloroplast mRNAs, but has a preference for *psbA* [9]. We validated this finding using an alternative detection technique, that is, microarray hybridization (RIP-chip) [38]. RIP-chip has the advantage over RIP-Seq that the co-precipiated RNA is directly labelled using a chemically activated dye without any further enzymatic steps. This avoids any experimental bias potentially introduced via enzymatic steps like reverse transcription, linker ligation, and PCR, or RNA size selections commonly used during library preparation for deep sequencing. Using the same antibody raised against a CP33B-specific peptide as in the RIP-Seq approach, we precipitated CP33B from chloroplast stroma. In line with our previous efforts, we found a clear preference of CP33B for the *psbA* mRNA (Figure 3a). The microarray used for these RIP-chip analyses consisted of PCR-generated probes that are mostly more than 500 nt in length. We next performed a fine-mapping analysis using a previously described oligonucleotide-based microarray [39] to confirm our findings. The oligo-RIP-chip verified the exceptional enrichment of *psbA* mRNA over other chloroplast mRNAs in CP33B precipitations (Figure 3b; Figure S1). In sum, *psbA* is the main target of CP33B.

### 2.3. CP33B Quantitatively Co-immunoprecipitates psbA mRNAs

The peak signal for *psbA* in CP33B RIP-chip experiments is remarkable given that this mRNA is the most abundant mRNA in chloroplasts. The RIP-chip can, however, only give relative insights into RNA quantification, which prompted us to use dot-blot, a semi-quantitative method to determine how much *psbA* mRNA is pulled down with CP33B. RNA from pellets and supernatants of CP33B precipitates from wt and from *cp33b* null mutant stroma preparations were blotted onto nylon membranes and probed with radiolabeled RNA probes. Importantly, equal fractions of supernatant and pellet volumes were blotted, which allows for a direct assessment of the efficiency of co-precipitation. Next to probes for several transcripts identified as targets of CP33B in RIP-chip analyses, we also used probes for two transcripts not enriched in an RIP-chip assay, *psbF* and the 23S rRNA. The hybridization confirmed that these two control RNAs do not co-precipitate with CP33B (Figure 3c). For all other transcripts tested, an accumulation in the precipitates from wild-type stroma was detected, but not in the precipitates of the mutants. Importantly, the signal for *psbA* in the pellet was far stronger than the residual signal in the supernatant. The ratio of the pellet to supernatant signals was far higher for *psbA* than for any other coding region analyzed (74-fold enrichment for the top *psbA* probe versus 12-fold enrichment for the top *psbD* probe). Thus, the vast majority of *psbA* transcripts in the stroma was associated with CP33B. About half of the *psaC* and *ndhK* transcripts co-precipitated with CP33B. The *rbcL* mRNA showed the lowest enrichment of all transcripts analyzed. As a caveat, it needs to be mentioned that RNA degradation occurs during the RIP-procedure. Thus, the enrichment observed reflects only CP33B’s association with the surviving RNA pool. Overall, the dot blot analyses confirmed the results of the RIP chip experiments and demonstrated that a majority of all *psbA* transcripts is associated with CP33B.

### 2.4. CP33B Prefers an RNA Sequence Motif in Vitro

**Figure 4:**
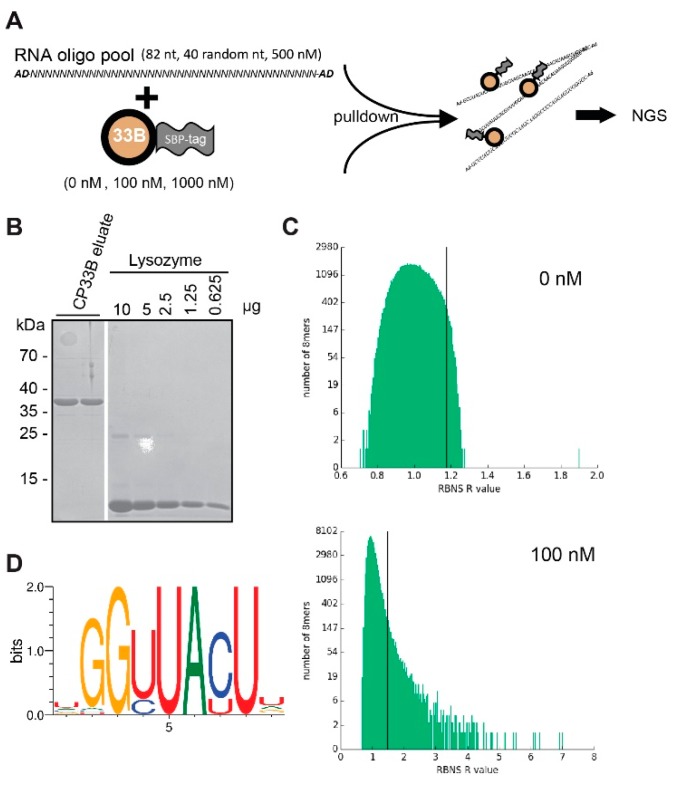
RBNS of CP33B. (**A**) Schematic overview of the RBNS experiment. (**B**) Coomassie stain of an analytical PAGE to determine the amount of SBP:CP33B in two replicate eluates after protease-based removal of the GST tag. The white line indicates where lanes irrelevant for this analysis were removed. (**C**) Stacked histogram showing the distribution of RBNS R values of all RNA 8mers in the CP33B experiment at a protein concentration of 0 and 100 nM, respectively. 8mers with R values that have z-scores equal or greater than two times the average are found on the right of the black lines. A log scale is used for the y axis. Please note the different scales for the X-axes between the two graphs. (**D**) CP33B consensus motif generated from all 8mers with a z-score ≥ 3 in the 100 nM library (detailed rules on motif generation found in [41]).

We next asked how the specificity of CP33B for *psbA* is determined. One explanation would be a target sequence motif that distinguishes the *psbA* mRNA from other chloroplast mRNAs. To test the RNA sequence preference of CP33B, we used the RNA-Bind-n-Seq (RBNS) assay (Figure 4A). RBNS is an in vitro method that allows the comprehensive mapping of RNA binding specificity [40,41]. It has considerable advantages over older in vitro methods like SELEX, which identify consensus motifs, but is biased towards the highest affinity motifs [42]. By contrast, RBNS tests affinities to the full spectrum of possible RNA sequences in a high-throughput manner. Being an in vitro technique, RBNS tests the direct interaction of the protein with RNA targets and avoids common biases of RIP-Seq and RIP-chip, which can potentially also enrich for RNAs indirectly tethered to CP33B via protein partners.

As a prerequisite for this analysis, we expressed recombinant CP33B with a combined N-terminal GST:SBP tag, but without the known signal peptide for chloroplast targeting, and purified it using a Glutathione matrix. The GST tag is subsequently cleaved off using the PreScission Protease and remains on the purification column, while pure SBP-CP33B is eluted and quantified using a lysozyme standard (Figure 4B). Two concentrations of recombinant SBP-CP33B (100 nM and 1000 nM) and a negative control (zero protein) were incubated with an input pool of random RNA 40mers. SBP-CP33B is pulled down using streptavidin-coated beads and the pulled-down RNA is used for library amplification and subsequently sequenced. The input random RNA pool is also sequenced to account for potential compositional biases. For each of the three protein concentrations (0, 100, and 1000 nM), enrichment (“R”) values were calculated for all *k*mers of selecetd lengths within the sequenced 40mers as the ratio of the frequency of the *k*mer in the sample pool to the frequency in the input pool. For CP33B, a large number of 6, 7, 8, and 9mer motifs had significant R values (as an example, the 8mer analysis in the 100 nM library is shown in Figure 4C). In our experiments, the highest R values were observed at a 100 nM CP33B concentration. At a higher protein concentration, the ranking of top-enriched *k*mers remains similar, even though the overall observed R values are lower. This is an expected behavior in RBNS experiments, since the top *k*mer targets are saturated at a certain protein concentration and secondary RNA targets become more co-precipitated as well, which lowers the overall R value. This observation and the ranking consistency between different libraries support the validity of our application of RBNS to CP33B. For a more detailed analysis, we chose 8mers, since single-RRM domains typically associate with 2–4 nucleotides and, thus, the two RRMs of CP33B may associate with up to eight nucleotides. All significant 8mers (with a Z-score ≥ 3) in the library with the highest enrichment (100 nM sample) were used to generate a consensus motif [41], which reflects the binding preference of CP33B (Figure 4D). We next asked whether the RBNS results reflect the preference of CP33B for the *psbA* mRNA. However, when performing sequence searches for derivatives of the top motif, there were dozens of hits throughout the chloroplast genome, but only one hit in the sense direction of the *psbA* coding region (Table S3). Possibly, the conditions used in our RBNS analysis might not adequately reflect the in vivo situation, preventing us from the finding the true in vivo target sequence. Alternatively, there could be other factors than simple sequence preferences in vivo that lead to the observed preference of CP33B for the *psbA* mRNA. Notably, at least three further RBPs, CP33C, SRRP1, and HCF173, were recently identified together with CP33B in precipitates of the *psbA* mRNA. Such additional RBPs could help CP33B to specifically recognize the *psbA* mRNA.

### 2.5. Membrane-bound CP33B is Also Associated with Chloroplast mRNAs

**Figure 5:**
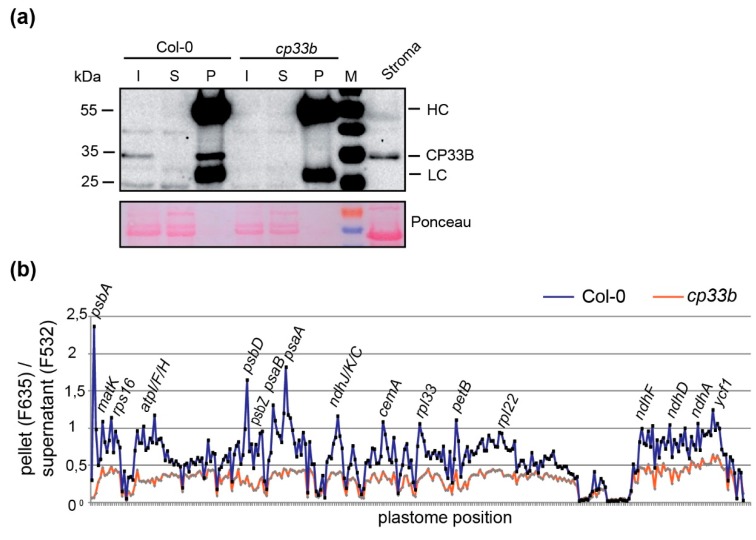
RNAs bound by membrane-localized CP33B. (**a**) Immunoprecipitation of CP33B from membranes. Immunoblot analysis of protein fractions after immunoprecipiation of CP33B from solubilized membranes of wt and *cp33b* null mutants. Relative to the volume of the input membrane fractions used (I) for the IPs, 1/20 of the supernatant (S) and 1/10 of the pellet (P) fractions were loaded. An aliquot of wt stroma from the same chloroplast preparation was analyzed as well, which shows the depletion of RuBisCO in the membrane fractions compared to the stroma fraction by Ponceau staining. Bands marked with HC and LC represent the heavy and light chains, respectively, of antibodies used for immunoprecipitation. (**b**) RIP-Chip analysis of membrane-bound CP33B. RNA from pellet and supernatant fractions of CP33B immunoprecipitations and control reactions were analyzed by microarray hybridization analogously to stroma RIP-Chips shown in Figure 3. The pellet/supernatant ratios (median (median of ratios)) were normalized to the sum of the median values of the ribosomal RNAs of the supernatants (median F532; Table S4). Ratios of fluorescence signals from co-precipitated RNA (F635) and unbound RNA (F532) for IPs from wt and *cp33b* mutant membranes were plotted against the probe positions on the chloroplast genome.

The RIP chip experiments shown in Figure 3 were performed with stroma material as input. Given that a part of the CP33B pool is associated with membranes, we performed RIP-chip on isolated membrane fractions that were solubilized prior to immunoprecipitation. Overall, RNA enrichment in the membrane RIP-chip is lower than in the stroma RIP-chip, which is indicated by the relatively small difference in enrichment values of the CP33B immunoprecipitation from wild-type membranes versus from control (*cp33b* mutant) membranes (Figure 5). Despite the lower signal, the analysis reveals that CP33B associates with multiple mRNAs also at membranes. Again, the top enriched mRNA is *psbA*, but the signal appears less dominant over secondary targets like *psbD/C, psaB/psaA, ndhJ/K/C, petB* and *atpF/H.* The 20 strongest enriched transcripts of the stroma RIP chip are also found among the strongest enriched mRNAs in the membrane RIP-chip (Tables S1 and S4). CP33B thus interacts with multiple and largely identical chloroplast mRNAs in the stroma as well as at membranes.

## 3. Discussion

### 3.1. CP33B is a Global RBP With a Clear Preference for and Ability to Sequester the psbA mRNA Pool

The RNA-binding spectrum of different RBPs can vary considerably. In higher plants, the PPR proteins, which are the most abundant family of RBPs in chloroplasts, have a small target spectrum of only one to a few transcript(s) [1]. In contrast, the cpRNPs interact similar to their nucleo-cytoplasmic relative hnRNP A1 [43] with a variety of RNAs, as shown for CP31A, CP29A, CP33A, and CP33B from Arabidopsis [7,8].

In this study, the RNA targets of CP33B from *Arabidopsis thaliana* were investigated in greater detail. We confirmed that CP33B associates with a larger number of chloroplast mRNAs. Similar to CP31A and CP29A [7], CP33B showed little or no enrichment of ribosomal RNAs and tRNAs. Also, several of its minor targets, like *atpH*, *psbC/D*, and *psaA/B* are also targets of the three other cpRNPs, CP31A, CP29, and CP33A, analysed before [7,8]. Possibly, the four cpRNPs are all part of common ribonucleoprotein particles. This extended redundant target range may, in part, explain the lack of a detrimental phenotype of *cp29a*, *cp31a,* and *cp33b* mutants, respectively, under normal conditions, where the loss of just one cpRNPs might be compensated by other members of the family.

Next to these commonalities in RNA targets, there are also striking differences for a selected low number of targets. Most strikingly, CP33B has a clear preference for a single mRNA, *psbA*, with an enrichment of 90% in precipitates as calculated from autoradiograph signals in the pellet versus supernatant fractions. The *psbA* mRNA is not, or is only a weak, target of CP33A, CP29A, and CP31A [7,8]. Vice versa, main targets of CP33A, like *psbF* or *rbcL*, of which more than 50% co-precipitate with CP33A [8], are either not (*psbF*), or are weak (*rbcL*), interaction partners of CP33B. How specificity is generated remains unclear. Our RBNS analysis uncovered a sequence motif as prime target that is not enriched in *psbA* versus other chloroplast transcripts. It does, however, prove that CP33B is capable of binding RNA by itself, without the need for auxiliary proteins, which is in line with previous electro mobility shift assays of cpRNPs [3,5,11]. Still, the specificity of binding is likely to be modulated by other proteins, as is frequently the case for RRM proteins [44]. For example, U2B, a structurally related RBP with two RRMs binds RNA in response to protein–protein interactions [45]. Candidates for protein interactions on the *psbA* mRNA are the two RBPs CP33C and SRRP1, which were co-purified in precipitations of the *psbA* mRNA, together with CP33B, although a direct interaction of these proteins was not demonstrated [9]. Another potential partner is HCF173, which is also known to be associated with *psbA* and to foster translation of this mRNA [9,35]. Speculatively, specificity for *psbA* might be generated by a larger complex of these three or more RBPs. The three proteins plus CP33B could together form an extended RNA-binding interface that would allow the specific recognition of *psbA*. CP33B is recruited by one or several of these RBPs and does not contribute to *psbA* specificity at all.

While presently unclear how specifically *psbA* is recognized by CP33B, it is remarkable that almost the entire *psbA* mRNA pool that survives the RIP procedure is associated with this protein. Given that *psbA* is the most abundant chloroplast mRNA, with an estimated 14.000 molecules per chloroplast [10], CP33B numbers should at least equal this amount, or, if we assume multiple target sites within the *psbA* mRNA, should be present in excess. *psbA*, like many of the mRNA targets of CP33B, encodes a photosynthetic membrane protein named D1. Such mRNAs, when translated, become tethered to membranes via nascent peptide chains being inserted co-translationally into the membrane [46]. CP33B binds predominantly within the coding region of *psbA,* according to our oligo-RIP-chip results. This is supported by RBNS data, which point to a sequence motif also found within the coding region of *psbA*. Binding to the coding region is likely facilitated by the absence of translating ribosomes that would be expected to remove obstacles like CP33B via their intrinsic helicase activity. Thus, translation would decrease the association of CP33B with *psbA* and this would occur on membranes. This idea is supported by our finding that CP33B prefers stromal *psbA* over membrane-tethered *psbA*. We therefore speculate that CP33B’s main role is centered on ribosome-free, stromal *psbA*. It is however puzzling that mutants of CP33B do not display defects in *psbA* accumulation or translation [9]. Redundancy or the lack of a specific stress signal might prevent the identification of the true function of CP33B for the *psbA* mRNA.

### 3.2. cpRNPs Interact With Their Target Transcripts via Multiple Binding Sites

As mentioned above, cpRNPs interact with many mRNAs, but where exactly does binding take place within a target transcript and can binding motifs possibly be identified? In order to answer these questions, the binding sites of CP33B should be described in more detail. In the past, the binding motifs of the splicing factor MatK [39] and various PPR proteins were deciphered with the help of an oligonucleotide microarray or comparable investigations (oligonucleotide probes on dot-blots of IP fractions) [47,48].

However, after performing CP33B RIP-chip analysis and hybridization on an oligonucleotide microarray, no enrichment of isolated short-sequence sections occurred, but rather an enrichment of almost all transcript sections of the target RNAs studied (*psbA, ndhB, psaA/psaB/rps14, ndhF* and *psbD/psbC*) was noted. This is in line with the finding that the intact *psbA* transcript can be recovered in CP33B IPs, as evidenced by RNA gel blot hybridization [9], whereas MatK only precipitated RNA fragments with a length of ~200 to ~500 nt and no full-length precursor transcripts [39]. A similar picture was observed using oligo RIP-chips analysis of CP29A and CP31A from Arabidopsis, as well as in vitro approaches for 28RNP from spinach [7,11]. 28RNP interacts with part of the coding regions, as well as the 3’ and 5’ UTRs of different mRNAs [11]. Together, these data could indicate that CP33B, as well as other cpRNPs, have multiple binding sites within their target transcripts. Such an RNA-association of cpRNPs across multiple binding sites is consistent with their general and global binding behavior, which is in contrast with the specific binding behavior of PPR proteins. Speculatively, such a multivalent interaction of cpRNPs with their RNA targets is functionally relevant, possibly for RNA protection against degradation, which is a main function of cpRNPs [7,8,10].

## 4. Materials and Methods

### 4.1. Plant Material

*Arabidopsis thaliana* cpRNP T-DNA insertion line *cp33b* (SK31607 from the Saskatoon collection) was obtained from the ABRC (Arabidopsis Biological Resource Center) and grown together with wt *A. thaliana* (ecotype Columbia-0) on soil with a 16-h light/8-h dark cycle at 23 °C.

### 4.2. Localization by Fluorescence Microscopy

A full-length cDNA sequence of CP33B was amplified by PCR using with oligonucleotides 33B.fw.xhoI.TP and 33B.rev.ncoI.cDNA and cloned via *Xho*I and *Nco*I as a translational fusion with GFP under the control of a 35S promoter. The PEND-dsRed construct, under constitutive control of the ubiquitin promoter (pUbi), was kindly provided by Dr R. Lorbiecke and Dr J. Kluth (University of Hamburg, Germany). Mesophyll protoplast preparation and transfection with CP33B:GFP- and PEND-dsRed-fusion constructs was performed as described [49]. A Zeiss 510 Meta confocal laser scanning microscope and the ZEISS LSM IMAGE BROWSER software were used for the detection and documentation of fluorescence signals.
**Oligonucleotide****Sequence**33B.fw.xhoI.TPATACTCGAGATGGCGGTTTTGGAAGC33B.rev.ncoI.cDNAATACCATGGCCACAATGTTTTCTTCG

### 4.3. Dot-Blot Analysis

Dot-blot production was described previously [8]. Radiolabeled RNA probes were prepared by in vitro transcription of PCR products containing a T7 promoter using T7 RNA polymerase in the presence of ^32^P-UTP.
**Oligo Name****Sequence****Target Gene**CK_atpH5‘_forGATTTAGATAGGGATTCGATTAG*atpH*CK_atpH5‘_T7_revGTAATACGACTCACTATAGGGTCCAGGTCCAATAGAAGCAAG*atpH*Ck_psbD_forCTATTTCGCTTTAGGGGGTTGG*psbD*Ck_psbD_T7_revTAATACGACTCACTATAGGGCAGCAATGGGACCAGAGAATGC*psbD*CK_rbcL_forGCAGCATTCCGAGTAACTCC*rbcL*CK_rbcL_T7GTAATACGACTCACTATAGGGCCACGTAGACATTCATAAACTGC*rbcL*CK-petB-forCGTCCAACCGTTACTGAAGC*petB*CK-petB-T7-revGTAATACGACTCACTATAGGGAATAGCGTCAGGTACACC*petB*CK-psbF-forGTCTGGAAGCACAGGAGAACG*psbF*CK-psbF-T7-revGTAATACGACTCACTATAGGGCAAAACGGCCTGTTATTAATGG*psbF*ndhBex1.rpCCGATGGAGAGAAGAACCTATG*ndhB*ndhBex1.T7GTAATCGACTCACTATAGGGTAGCCAAGAGAAACCATGAACC*ndhB*ndhK.rpCTATGGCCGCTTCTTTATGG*ndhK*ndhK.T7GTAATCGACTCACTATAGGGATTTCCAATATGCGGACTGC*ndhK*psbA.rpCATTCATTGCTGCTCCTCCAG*psbA*psbA.t7neuGTAATCGACTCACTATAGGGATTCCTAGAGGCATACCATCAG*psbA*psbC.rpAGTGGCCCATTTTGTACCTG*psbC*psbC.T7GTAATCGACTCACTATAGGGCCCCCAAAGGGAGATTTTAG*psbC*rrn23.rpCCTAGATGGCGAGAGTCCAGrrn23rrn23.T7GTAATCGACTCACTATAGGGAAGACTCGCTTTCGCTACGrrn23

### 4.4. Immunoblots and Antibody Production

The production and specificities of the CP33B antisera were reported previously [9]. The antisera to PsaD and RuBisCO were obtained from Agrisera (cat. no. AS09 461; AS03 037; Vannas, Sweden). Immunoblots were carried out using standard procedures. Quantification of CP33B in membrane and stroma fractions of chloroplasts was carried out as follows. Chloroplasts were purified from Col-0 plants, disrupted by hypotonic buffer and by pulling through a 0,4 mm gauge syringe and then separated into soluble (stroma) and insoluble (membrane) fractions via centrifugation (21.000 x g, 4°C, 30min). A total of 10% (vol/vol) of both, stroma and membrane fraction were analyzed by Western blot. The signals were quantified with the Image Lab Software (Bio-Rad).

### 4.5. RIP-chip Analysis

CP33B was immunoprecipitated from Arabidopsis chloroplast stroma and co-precipitating RNAs were purified and hybridized to a whole-chloroplast-genome tiling array or a oligonucleotide array, respectively, as previously described [7]. The hybridized microarray was washed, scanned and analyzed as described [8]. RIP-chip of CP33B from membrane fractions was performed on membranes prepared from isolated chloroplasts. The membranes were washed five times, solubilized with 1% NP 40 (NonidetP 40) for 15 min on ice and centrifuged (10min, 20,000 g, 4 °C) to remove unsoluble matter. The dissolved membranes were diluted with 1 vol CoIP buffer (150 mM NaCl, 20 mM Tris-HCl (pH 7,5), 1 mM EDTA, 2 mM MgCl_2_, 0,5% (v/v) NonidetP-40, 5 μg/mL Aprotinin) and incubated with anti CP33B antisera. All further procedures including data evaluation were carried out analogous to that of the Stroma RIP-chip.

### 4.6. RBNS Analysis

#### 4.6.1. Cloning of GST:SBP:CP33B

A streptavidin-binding protein (SBP) tag was introduced into the *pGEX-6P-1* vector downstream of the GST tag and the PreScission cleavage site of the vector via *Bam*HI and *Eco*RI restriction sites. The resulting vector was named *pGEX-SBP*. The coding sequence of CP33B was amplified from cDNA without the predicted signal peptide and cloned into the *pGEX-SBP* vector via *Mfe*I and *Xho*I sites using the following oligonucleotides.
**Oligonucleotide****Sequence**CP33BXhoIrev AATTCTCGAGTCACTCCACAATGTTTTCTTCGG MfeICP33BforAATCAATTGGTCTCCGTACTCTGTTCCGTC

#### 4.6.2. RNA Oligo Production

The RBNS random RNA input for the RNA Bind-n-Seq experiment was prepared by in vitro transcription of the RBNS T7 template, a DNA oligo containing a 40mer random sequence flanked by primer sites for the Illumina adapters and a T7 promoter sequence [50]. To produce the dsDNA for a more optimal transcription from the T7 polymerase, the T7 promoter oligo was first attached to the RBNS T7 template region corresponding to the T7 promoter. For this purpose, 3 μL of each of the 100 μM oligo samples were heated in water at 65 °C for 5 min and then cooled at room temperature for 2 min. Polymerization of the second DNA strand was performed within 15 min at 25 °C in the presence of 33 μM dNTPs, 1x NEB2 buffer and 11 U DNA Polymerase I Large (Klenow) fragment in a 50 μL volume reaction. The reaction was then stopped at 75 °C for 20 min with 10 mM EDTA.

The RBNS Input Oligo Pool was transcribed with T7 RNA Polymerase. DNA was removed using 2 U TURBODNase (Life Technologies) for 5 min at 37 °C. The reaction was stopped by adding 1.5 μL EDTA 0.5 M and incubation at 75 °C for 10 min. The in vitro transcribed RNA were purified by gel extraction and concentrated by ethanol precipitation.
**Oligonucleotide****Sequence**RBNS T7 Template CCTTGACACCCGAGAATTCCA -(N)40- GATCGTCGGACTGTAGAACTCCCTATAGTGAGTCGTATTA RNA PCR (RP1) AATGATACGGCGACCACCGAGATCTACACGTTCAGAGTTCTACAGTCCGACGATC RT primer GCCTTGGCACCCGAGAATTCCA T7 Promoter Oligo TAATACGACTCACTATAGGG 

#### 4.6.3. RBNS Assay

Two different concentrations of SBP-CP33B, 100 and 1000 nM, were equilibrated in 250 μL binding buffer (25mM Tris-HCl pH 7.5; 150 mM KCl; 3mM MgCl2; 0.01% tween; 1 mg/mL BSA; 1 mM DTT) at 22 °C for 30 min. In addition, a sample with binding buffer only (zero protein sample) was incubated in the same way. The RBNS Input Random RNA was then added to a final concentration of 0.5 μM and incubated for 3 h at 22 °C. Subsequently, streptavidin-coated magnetic beads (Invitrogen) were added and the bead-sample mixture was incubated for 1 h at room temperature. After magnetic separation, the beads were washed with 1 mL Wash Buffer (25mM Tris-HCl pH 7.5; 150 mM KCl; 60 ug/mL BSA; 0.5 mM EDTA; 0.01% tween) and then incubated at 70 °C for 10 min in 100 μL Elution Buffer (10mM Tris-HCl pH 7.0, 1mM EDTA, 1%SDS). RNA from the eluates were purified using the RNA Clean & Concentrator Kit by Zymo Research (Irving, USA). Half of the purified RNA from each protein concentration and the input RNA pool was reverse-transcribed using the ProtoScript II reverse transcriptase using the RT primer and subsequently amplified by PCR using the Q5 High-Fidelity DNA polymerase and the RP1 primer (see above) plus barcoded primers. The library was sequenced on a NextSeq 500 (Illumina) at LGC (Berlin). For analysis and motif detection, we used the published bioinformatic pipeline [40,41,51]
**Sample****NEXTflex Barcode Primer Sequence**Input 5 ′-CAAGCAGAAGACGGCATACGAGAT- CGTGATGTGACTGGAGTTCAGACGT- GTGCTCTTCCGATC-s-T-3 ′ 0 protein sample5 ′- CAAGCAGAAGACGGCATACGAGA- TGTAGCCGTGACTGGAGTTCAGACGT- GTGCTCTTCCGATC-s-T-100 nM CP33B 5 ′-CAAGCAGAAGACGGCATACGAGA- TCTGATCGTGACTGGAGTTCAGACGT- GTGCTCTTCCGATC-s-T-3 ′ 1000 nM CP33B 5 ′-CAAGCAGAAGACGGCATACGAGAT- AAGCTAGTGACTGGAGTTCAGACGT- 

## Supplementary Materials

The following are available online at https://www.mdpi.com/2223-7747/9/3/367/s1, Figure S1: Details of CP33B oligo-RIP-chip analysis, Table S1: RIP-Chip data for Fig. 3a, Table S2: Oligo RIP-Chip data for Fig. 3b and Fig. S1, Table S3: summary of sites in the chloroplast genome of Arabidopsis that correspond to the top motif of the RBNS analysis, Table S4: RIP-chip data for Fig. 5B (CP33B RIP-chip from membranes).
