# Supplementary material for: The Chloroplast Ribonucleoprotein CP33B Quantitatively Binds the psbA mRNA
Source: Plants (Basel). 2020 Mar 17;9(3):367. doi: 10.3390/plants9030367 (PMC7154868; doi:10.3390/plants9030367)
Supplement: Supplementary file 1 [file plants-09-00367-s001.pdf]

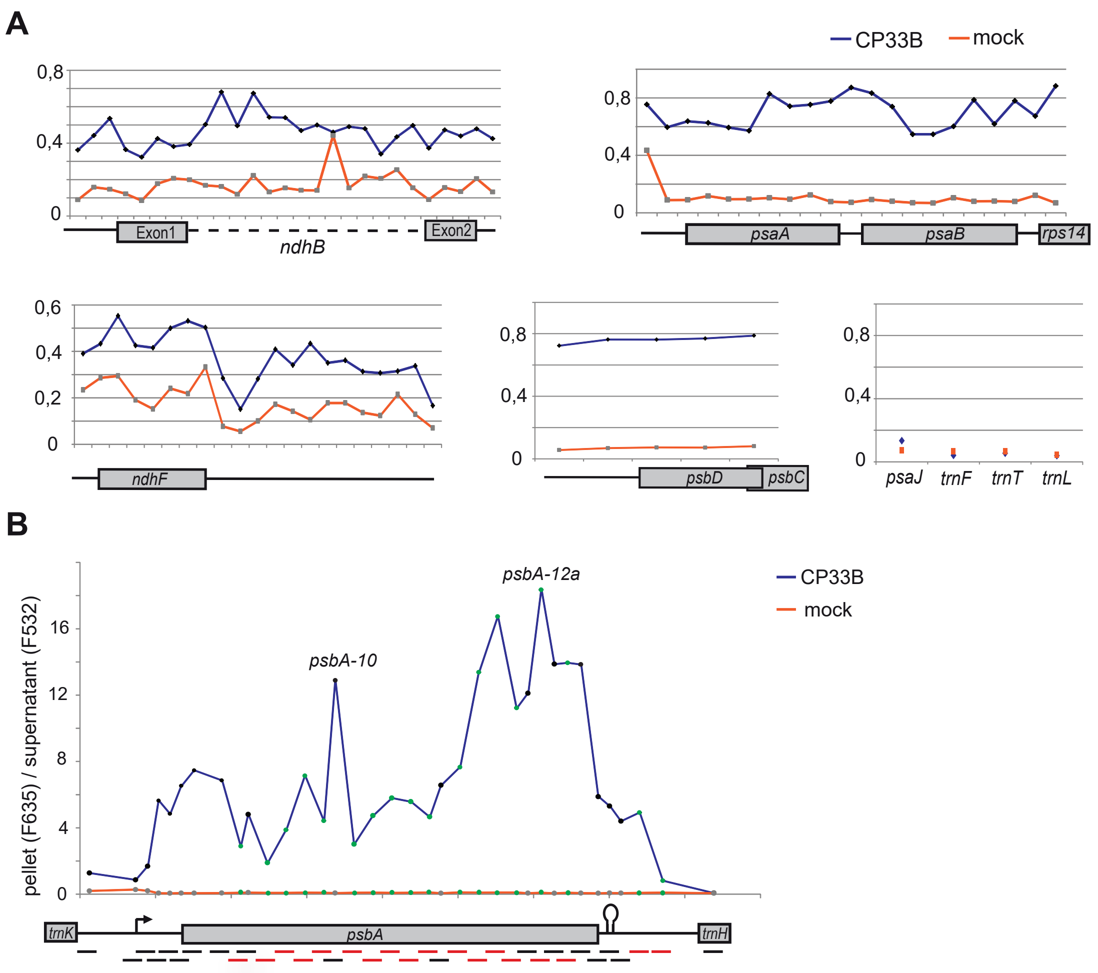

Suppl. Figure 1: Details of CP33B oligo-RIP-chip analysis

A) Enlarged view of the RIP-chip results of individual transcripts from Fig. 3B, except for *psbA* (see B). Grey bars represent coding regions, black lines represent non-coding regions and the dashed line represents the *ndhB* intron. Sequences for *psaJ, trnF, trnT* and *trnL* were used as control oligonucleotides (lower right figure).

B) Enlarged view of the oligonucleotide RIP-chip analysis for the interaction of CP33B with *psbA*. Since further *psbA* oligonucleotides were added to the microarray at a later stage for a more precise breakdown, some enrichment values were formed from only two biological replicates of CP33B IP and one replica with pre-immune serum as controls (shown in green). Grey bars represent the coding regions (*trnK, psbA* and *trnH*) and black lines in between represent the non-coding regions. The transcription start for *psbA* (arrow) and a stem-loop in the 3'UTR are shown. The positions of the oligonucleotides used in the microarray are symbolized by short black/red lines below the gene map.

Supplemental Table 1: RIP-Chip data for Figure 3a

Name			Median(F532 Median - B532)		Median (Median of Ratios (635/532))		Diff. Enrich. [2]
	Spot Count CP33B [1]	Spot Count control [1]	CP33B	control	CP33B	control	(CP33B/ control)
H-psbA	36	36	4896	10997	0.865	0.26964	3.207981012
psbA	36	35	2968	10489	19.294	0.2604	74.093702
psbA-Kex2	36	36	999.5	6256.5	8.6185	0.27636	31.18577218
Kex2-matK	36	36	775	815	0.397	0.4368	0.908882784
matK-1	35	36	507	992.5	1.449	0.59556	2.433004231
matK-2	35	32	126	297.5	0.738	0.85596	0.862189822
Kint	30	24	160	2425.5	0.8025	0.3024	2.653769841
Kint-Kex1	36	25	135.5	250	1.3955	0.46704	2.987966769
IG_K-rps16	35	36	334	877.5	1.507	0.80724	1.866854963
rps16ex2	35	36	629	1171	1.006	0.41748	2.409696273
rps16ex2-in-ex1	30	30	173	268	1.884	1.17936	1.597476597
rps16in-1	27	22	153	2763.5	0.57	0.10752	5.301339286
rps16in-2	36	30	416	686	0.963	0.60144	1.601157223
rps16ex2-IG	27	27	301	341	0.538	1.28016	0.420259968
Q	36	36	7156	9036.5	0.0505	0.3024	0.166997354
psbK	36	36	2897.5	6257	0.461	0.24528	1.879484671
psbI-S	36	36	1721.5	3708.5	0.219	0.28224	0.775935374
IG-psbI-G-1	36	36	18778.5	36461	0.0445	0.21084	0.21106052
IG-psbI-G-2	36	36	2310.5	3966	0.3075	0.26292	1.16955728
Gex1-in	36	36	2531	3227.5	0.456	0.28644	1.591956431
in-Gex2	36	36	2946	6737	0.2665	0.25452	1.047068993
Gex2-R	36	36	1160.5	2464.5	0.5935	0.28056	2.115412033
atpA-1	35	28	230	320	0.61	0.80472	0.758027637
atpA-3	36	36	355.5	831.5	1.923	0.651	2.953917051
atpA-atpFex2	36	36	270.5	585	2.102	0.58716	3.579944138
atpFex2-in	36	36	857.5	2720	2.322	0.3948	5.881458967
atpFex2-in-ex1	36	36	1526.5	5452.5	1.525	0.26712	5.709044624
atpFin-ex1	36	36	864	2863	1.7555	0.35952	4.882899421
atpFex1-IG	36	36	1602	4978.5	3.223	0.30828	10.45478137
atpH	36	33	239	702	1.7405	0.68544	2.539244865
atpH-IG	36	36	3138.5	6641	4.7255	0.25452	18.56632092
IG-atpI	32	36	282	1146	2.1335	0.48132	4.432602011
atpI	36	33	259.5	822	3.1145	0.672	4.634672619
atpI-rps2	36	36	1058	3704	2.435	0.37212	6.54358809
rps2	36	24	167.5	1488.5	0.4105	0.56364	0.728301753
rps2-IG	36	21	81.5	5351	1.8615	0.0672	27.70089286
rpoC2-1	27	24	127	1139.5	1.515	0.76776	1.973272898
rpoC2-3	36	27	111	5268	3.1585	0.63168	5.000158308
rpoC2-4	36	20	131	391	1.831	0.04788	38.24143693
rpoC2-5	30	21	146.5	6858	0.687	0.0588	11.68367347
rpoC2-6	27	24	129	4321	1.357	0.43176	3.142949787
rpoC2-7	24	24	113	2622	1.1505	0.36624	3.1413827

rpoC2-rpoC1-1	21	25	2862	7426	0.111	0.48048	0.231018981
rpoC1ex1-1	36	27	3417	419	1.51	0.80976	1.864750049
rpoC1ex1-in	36	24	1291.5	2211	1.639	0.40488	4.048113021
rpoC1ex1-in-ex2	36	24	166.5	255	1.362	0.1848	7.37012987
rpoC1in	36	32	146	2539.5	1.869	0.91056	2.052583026
rpoC1in-ex2	29	27	97.5	2744.5	2.772	0.88872	3.119092628
rpoC1-rpoB-1	30	27	148.5	2330.5	1.5755	1.01808	1.547520824
rpoC1-rpoB-2	36	30	265	378	1.064	0.6048	1.759259259
rpoB-1	36	36	142	294	1.5725	0.91812	1.712739076
rpoB-2	27	23	140.5	276	1.6	0.14112	11.33786848
rpoB-3	30	24	161.5	417	0.844	0.51576	1.63642004
rpoB-4	36	36	211	445	2.82	1.20372	2.342737514
rpoB-IG	24	27	160	3317	0.73	1.56744	0.465727556
IG rpoB-C	36	27	94.5	3436.5	2.3055	1.08192	2.130933895
IG-C	36	36	734.5	675.5	0.0715	0.2814	0.254086709
C-IG	36	36	1035.5	179	0.6275	0.37464	1.674941277
IG-petN	36	36	135	410	0.3795	0.27972	1.356713857
petN-IG	36	36	6955.5	11301.5	0.6235	0.4914	1.268823769
psbM	24	20	563.5	1030	0.2075	0.08652	2.398289413
IG psbM-D-1	36	36	2043.5	3477	0.413	0.37968	1.087758112
IG psbM-D-2	36	34	668	1012	0.7685	0.8316	0.924122174
D	36	33	587.5	3066.5	0.1735	0.32928	0.526907191
Y	35	36	984.5	1112	0.575	0.42504	1.352813853
E	36	36	183.5	297.5	0.4675	0.32928	1.419764334
IG E-T	27	24	1276	3006	1.345	0.75936	1.771228403
T	36	36	593	973.5	0.2325	0.45276	0.513517095
IG T-psbD-1	36	36	2569	3560.5	1.4555	0.48216	3.018707483
IG T-psbD-2	36	36	81	1691.5	1.761	0.21924	8.032293377
psbD-1	36	36	991	1134	4.011	0.32424	12.37046632
psbD-2	36	36	630.5	1606.5	3.302	0.40152	8.223749751
psbD-psbC	36	36	2249.5	5629.5	2.3455	0.39564	5.928369225
psbC-1	36	36	1811.5	4857	1.6015	0.27132	5.902624208
psbC-2	36	36	2276.5	3569.5	2.599	0.26292	9.885136163
psbC-S	36	36	327.5	2312	1.2265	0.27888	4.397948939
S-psbZ	36	36	2016	3570.5	2.1935	0.33852	6.479676238
IG psbZ-G	36	36	3267	7068	0.3125	0.31836	0.981593165
G-fM	36	36	2211.5	4579.5	0.174	0.3696	0.470779221
fm-rps14	36	36	1089	2343.5	0.144	0.3024	0.476190476
rps14-psaB-1	36	36	1453.5	2553	1.0915	0.28308	3.85580048
rps14-psaB-2	36	36	1588	1865	0.95	0.31752	2.991937516
psaB-1	36	36	3493	4891.5	2.3715	0.294	8.066326531
psaB-2	36	36	1719.5	4021	1.2615	0.41244	3.058626709
psaB-psaA-1	36	36	961	2540.5	1.9165	0.58968	3.250067833
psaB-psaA-2	36	27	3030	5264.5	1.588	0.32424	4.897606711
psaA-1	36	36	422	1486	1.7435	0.31836	5.476504586
psaA-2	35	32	333	1004	1.332	0.65268	2.040816327
psaA-3	36	36	201	2173	2.101	0.30324	6.928505474
psaA-IG-1	36	36	1816.5	2713.5	1.5685	0.31332	5.006064088
psaA-IG-2	27	24	307	958.5	1.992	0.61656	3.230829116
IG-ycf3ex3	36	36	1206.5	3277	1.096	1.49436	0.733424342

ycf3ex3-in2	24	33	1432.5	2560.5	1.0835	1.23984	0.873903084
ycf3ex3-in2-ex2	36	36	137	2233	0.821	0.5628	1.458777541
ycf3in2	33	27	187.5	242	1.977	0.80136	2.467056005
ycf3ex2	36	27	702	296	2.2975	0.85512	2.686757414
ycf3ex1	36	33	342.5	577	0.7745	0.7644	1.013212977
S-GGA	36	36	202	372	0.0665	0.2478	0.268361582
rps4-1	32	23	124	473	0.9155	0.07392	12.38501082
rps4-2	32	33	969	5234	0.979	1.04664	0.93537415
T-UGU	35	36	160.5	311	0.45	0.55524	0.810460341
T-IG	36	36	3436.5	5409	0.058	0.23184	0.250172533
L-UAA ex1-in	36	36	161.5	4510	0.1505	0.27888	0.539658635
L-UAA in-ex2	36	36	207.5	363	0.1255	0.231	0.543290043
L-UAA ex2-IG	36	36	512	679	0.1715	0.26376	0.650212314
F-GAA-1	36	36	3596	5195.5	0.0715	0.24948	0.28659612
F-GAA-2	36	36	8786	11786	0.159	0.29904	0.531701445
ndhJ	36	36	6748.5	10830	1.228	0.48132	2.551317211
ndhJ-ndhK	36	36	3352.5	6778.5	2.1165	0.5838	3.625385406
ndhK	36	30	3395.5	6486	3.11	0.34524	9.008226162
ndhK-ndhC	36	36	2867	4286	3.102	0.399	7.77443609
ndhC-IG	36	36	357.5	923.5	3.4985	0.31752	11.01820358
IG ndhC-V-UAA	36	24	208	616.5	1.07	0.42	2.547619048
Vex2-in	35	27	217.5	2014.5	0.684	0.23352	2.929085303
Vin-ex1	36	36	453	1585	0.1415	0.34944	0.404933608
Vex1-atpE	36	36	754	1863	0.0975	0.4578	0.212975098
atpE-atpB	35	36	264	3357	0.397	0.45948	0.864020197
atpB-1	35	36	246	7685	0.465	0.48972	0.949522176
atpB-2	36	36	1335.5	2004	0.9215	0.71484	1.289099659
atpB-3	36	36	2371	4049.5	0.693	0.7644	0.906593407
atpB-rbcL	36	36	540	950.5	0.4615	0.38556	1.196960266
rbcL-1	36	36	1053	1727.5	1.005	0.399	2.518796992
rbcL-2	36	36	390.5	574.5	0.9705	0.39816	2.437462327
rbcL-3	36	36	892	1489	1.3665	0.44184	3.092748506
IG rbcL-accD	36	36	1038.5	2157.5	0.4545	0.5124	0.887002342
accD-1	31	34	2738.5	4672.5	0.921	0.68796	1.338740624
accD-2	36	36	2596	4463.5	1.122	0.61572	1.82225687
accD-3	36	33	4543	6965	0.899	0.6468	1.389919604
IG accD-psaI	36	36	437.5	762	0.2705	0.23856	1.133886653
psaI	36	36	321	332.5	1.481	0.20328	7.285517513
ycf4-1	36	34	366	582	1.182	0.294	4.020408163
ycf4-2	36	33	243.5	557	0.7345	0.24696	2.974165857
cemA-1	36	33	1148	4940.5	1.1075	0.65688	1.686000487
cemA-2	36	36	449.5	5280.5	0.995	0.39228	2.536453554
cemA-3	36	36	1268.5	9907	0.843	0.51996	1.62127856
petA-1	36	32	296	5114	0.6795	0.2604	2.609447005
petA-2	36	36	433	553	0.701	0.546	1.283882784
petA-3	36	36	673	2189.5	0.5895	0.59304	0.994030757
IG petA-psbJ	36	33	910	1201.5	0.47	0.47544	0.988557967
psbJLF	36	36	275	3724	0.217	0.39816	0.545007032
psbFE	36	36	1724	1813	0.3815	0.25116	1.518952062
IG psbE-petL-1	36	36	1089.5	1773.5	0.3305	0.28056	1.178001141

IG psbE-petL-2	36	36	1063.5	1258	0.732	0.25872	2.829313544
IG psbE-petL-3	36	36	6966.5	7125	0.6015	0.44772	1.3434736
petL	36	36	3610.5	5653	0.3735	0.45444	0.821890679
petG-W-CCA	36	36	2485	3945.5	0.2565	0.29064	0.882535095
P-UGG	36	36	434.5	3612	0.0785	0.42084	0.186531699
psaJ	35	36	525.5	910	0.113	0.38136	0.29630795
IG psaJ-rpl33	36	36	1964	3208.5	0.439	0.378	1.161375661
rpl33	36	36	1393.5	2372	0.6425	0.63168	1.017128926
rpl33-rps18	36	36	5175.5	5926	0.622	0.504	1.234126984
rps18	35	35	5103	8587	0.785	0.27888	2.814830752
rpl20	32	32	1559	2512.5	0.5285	0.24864	2.125563063
rpl20-IG	33	35	848	1164.5	0.652	0.40488	1.610353685
in1-rps12ex1	33	36	1197.5	963	0.736	0.27132	2.712664013
rps12ex1-clpPex3	36	36	349	3102	0.6255	0.38976	1.604833744
clpPex3-in2	35	36	207.5	4146.5	0.464	0.73164	0.634191679
in2-clpPex2	36	35	224	988	0.5975	0.77448	0.771485384
clpPex2-in1	36	36	497	2757	0.484	0.75348	0.642352816
clpPin1	36	36	1246.5	1671.5	0.4755	0.36204	1.313390786
clpPex1	36	36	606	906.5	1.173	0.24108	4.865604778
psbB-1	36	36	316	407	2.1125	0.26964	7.834520101
psbB-2	36	36	576.5	613.5	0.7715	0.27804	2.774780607
psbBTN	36	36	721.5	1509.5	0.9385	0.36372	2.580281535
psbNH	36	36	913	3189.5	0.246	0.42252	0.58222096
psbH-petBex1-in	36	36	1915.5	4513	0.9915	0.36792	2.694879322
petBin	36	36	582	2669.5	1.6435	0.45276	3.629958477
petBin-ex2	36	36	597.5	4179	2.5625	0.23604	10.85621081
petBex2	36	36	2698.5	3685.5	1.5335	0.52752	2.906998787
petBex2-petDex1-in	36	36	458.5	1613	0.9835	0.44688	2.200814536
petDin	36	36	711	1521.5	0.753	0.31668	2.377794619
petDex2	36	36	1322.5	3166	1.73	0.30912	5.596532091
petD-rpoA	36	36	1036.5	1403	0.473	0.43428	1.089159068
rpoA	36	36	1492	2849	0.5705	0.34944	1.632612179
rpoA-rps11	33	34	1268	2270.5	0.605	0.52836	1.145052616
rps11-rpl36	36	24	1180	1993.5	0.948	0.14112	6.717687075
IG rpl36-rps8	36	34	1583.5	2513.5	0.8475	0.52164	1.62468369
rps8	33	27	2664.5	4792	0.684	0.4452	1.53638814
rps8-rpl14	36	36	250	623	0.81	0.40908	1.980052801
rpl14	36	36	372	4212.5	1.0635	0.40992	2.594408665
rpl16ex2	36	35	207.5	497.5	0.9765	0.33264	2.935606061
rpl16in-1	35	36	199	972	0.96	0.21252	4.517221909
rpl16in-2	24	24	384	888	0.926	0.1428	6.484593838
rpl16in-ex1	36	33	503	961	1.122	0.252	4.452380952
rps3-1	33	35	350.5	7197	0.848	0.18648	4.547404547
rps3-2	35	33	292	5128	0.937	0.2604	3.598310292
rpl22	36	36	668.5	4204.5	1.103	0.26124	4.222171184
rps19	35	36	195.5	6589	0.948	0.29736	3.188054883
rps19-rpl2ex2	36	36	196	7091	1.9835	0.27216	7.287992357
rpl2ex2-in	36	36	176	2227	2.017	0.24864	8.112129987
rpl2ex2-in-ex1	36	36	246.5	1590.5	3.1065	0.48048	6.46540959

rpl2in-ex1	36	36	415	1864	0.5765	0.29904	1.927835741
rpl2ex1	36	36	511	2374	1.733	0.39228	4.417762822
rpl23-I-CAU	36	34	351	8055	1.796	0.9072	1.979717813
I-CAU-ycf2	36	36	589	1198.5	0.138	0.41832	0.329890993
ycf2-I-CAU	36	36	560	1020	0.176	0.3024	0.582010582
ycf2-1	36	26	4449.5	6352.5	0.9375	0.86268	1.086729726
ycf2-9	36	28	262	433	1.81	0.6132	2.951728637
ycf2-3	33	29	429.5	1749.5	1.238	0.75768	1.633935171
ycf2-4	36	30	312	523.5	1.1375	0.441	2.579365079
ycf2-5	34	29	1879.5	2150	1.276	0.75768	1.684088269
ycf2-6	36	31	285	321	1.9435	0.6636	2.928722122
ycf2-7	36	32	134	275	0.9835	0.5922	1.660756501
ycf2-8	36	33	264.5	710	0.573	0.70224	0.815960355
ycf2-IG	36	31	216.5	345	0.698	0.25536	2.73339599
IG-L-CAA	36	30	249.5	533	0.8115	0.30828	2.632347217
L-CAA	33	30	373.5	664	0.57	0.26712	2.133872417
ndhBex2-1	36	36	421	477	0.76	0.40656	1.869342778
ndhBex2-2	36	33	209.5	9832	0.846	0.30408	2.782162589
ndhBex2-in	36	33	306.5	1580.5	1.226	0.2772	4.422799423
ndhBex2-in-ex1	36	36	259	5917.5	1.274	0.48636	2.619458837
ndhBin-ex1	33	27	373	1692.5	0.864	0.3696	2.337662338
ndhBex1-1	36	36	387	1136	1.405	0.62496	2.248143881
ndhBex1-2	36	36	275.5	6025	1.338	0.32844	4.073803434
IG-rps7	36	30	462.5	865.5	0.573	0.35364	1.620291822
rps7-rps12ex3	36	36	241	1306	0.8185	0.30744	2.662308093
rps12in2-ex2	36	36	446.5	515.5	1.649	0.70308	2.34539455
rps12ex2	36	36	750.5	3298.5	0.9165	0.52332	1.751318505
rps12in1	36	36	293.5	2545	0.892	0.42924	2.078091511
IG rps12-V-GAC-1	36	31	5859	8411	3.0565	0.90216	3.387979959
IG rps12-V-GAC-2	35	33	381	933	2.3	1.0416	2.208141321
IG rps12-V-GAC-3	36	28	633.5	1296.5	2.0415	0.85764	2.380369386
IG rps12-V-GAC-4	33	33	1222	1470	1.925	0.82656	2.328929539
IG rps12-V-GAC-5	35	29	158.5	483	1.553	0.72912	2.129964889
V-GAC	36	36	232	359	0.3415	0.32928	1.037111273
V-GAC-16S	36	36	172	492.5	0.098	0.30996	0.316169828
16S-1	36	36	503	445	0.0925	0.28896	0.320113511
16S-2	36	36	265	585	0.0955	0.2898	0.329537612
16S-3	36	36	1464	3246.5	0.083	0.31584	0.262791287
16S-4	36	36	23984.5	30137	0.0785	0.26796	0.292954172
16S-5	36	36	27257	48301	0.2145	0.27552	0.778527875
I-GAU ex1	36	36	15318.5	35822.5	0.62	0.2898	2.139406487
I-GAU ex1-in	24	36	24784.5	28595	3.1935	0.357	8.945378151
I-GAU ex2	36	36	21282.5	34325.5	0.97	0.28056	3.457370972
I-GAU-A-UGC	36	36	10051.5	20862.5	0.314	0.3696	0.8495671
A-UGC ex1-in	36	36	8104.5	14494	0.574	0.36792	1.560121766
A-UGC -in	36	36	2763.5	6826	0.588	0.41412	1.419878296
A-UGC ex1	36	36	7812.5	9685	0.451	0.32424	1.390944979
A-UGC-23S	36	36	2096	4109.5	0.1625	0.22008	0.738367866
23S-1	36	36	1198.5	3319.5	0.168	0.20328	0.826446281
23S-2	36	36	2340	3639	0.1475	0.22092	0.667662502

23S-3	36	36	2332.5	4745	0.177	0.23856	0.74195171
23S-4	36	36	23287	39978	0.191	0.20244	0.943489429
23S-5	36	36	19590	36516	0.1565	0.23016	0.679961766
23S-6	36	36	7680.5	21798	0.1625	0.25284	0.64269894
23S-7	36	36	24805.5	41495	0.175	0.24024	0.728438228
23S-8	36	36	35717	45359	0.1925	0.23604	0.815539739
23S-9	36	36	20270	36888	0.0575	0.20328	0.282861078
4.5S	36	36	12367	25611.5	0.0785	0.23688	0.331391422
R-ACG-1	36	36	29521.5	43793	0.283	0.27972	1.011726012
R-ACG-2	36	35	13801	13724.5	1.711	0.33768	5.066927268
N-GUU-1	35	36	20210.5	31881.5	0.634	0.30072	2.108273477
IG-ycf1	36	36	12483	22290	0.17	0.3108	0.546975547
3'ndhF(ycf1)	36	36	5159	8611.5	1.4305	0.50316	2.843032038
ndhF-1	36	36	364	2107	1.1915	0.315	3.782539683
ndhF-2	36	36	754	4614	0.8035	0.35868	2.240158358
ndhF-3	36	36	3847.5	6334.5	1.19	0.32172	3.698868581
ndhF-4	36	36	1211	2107.5	2.312	0.54012	4.280530253
ndhF-5	36	36	740.5	1149	0.9015	0.27972	3.222865723
ndhF-IG	36	36	997	1381.5	0.5955	0.32004	1.860704912
IG ndhF-rpl32	33	30	637	1399.5	0.341	0.3192	1.068295739
rpl32	36	33	497	1010	0.1315	0.19656	0.669006919
IG rpl32-ccsA	27	20	4689.5	9247.5	1.933	0.04368	44.253663
IG-ccsA	33	33	365.5	1137.5	0.75	0.40488	1.852400711
ccsA	36	36	210	539.5	3.9475	0.35616	11.0835018
ccsA-ndhD	33	24	1031	1707	2.977	0.26544	11.21534057
ndhD-1	36	36	90	5625	1.1665	0.48216	2.419321387
ndhD-2	36	26	280	530	1.4445	0.54096	2.670252884
ndhD-psaC	36	36	1045	2450.5	1.625	0.32424	5.011719714
psaC-ndhE	36	36	101	3188	0.575	0.26544	2.166214587
ndhE	24	36	579.5	820.5	1.537	0.30576	5.02681842
IG-ndhG	36	36	609.5	833.5	0.5445	0.23772	2.290509844
ndhG	36	36	1153	3314	0.977	0.3612	2.704872647
ndhG-ndhI	36	36	4404	10496	0.792	0.2352	3.367346939
ndhI	36	36	1457.5	1946.5	1.4315	0.44436	3.221487083
ndhI-ndhAex2	36	36	1628	3493	1.6075	0.53676	2.994820777
ndhAex2-in	36	36	1328.5	2354	1.7155	0.46032	3.726755301
ndhAex2-in-ex1	36	35	1880	4248.5	0.6435	0.4452	1.44541779
ndhAin-1	36	34	545	852	0.8755	0.48972	1.787756269
ndhAin-2	36	36	335	877.5	0.819	0.47208	1.734875445
ndhAin-ex1	36	36	593	772.5	1.6985	0.54096	3.139788524
ndhAex1-ndhH	36	36	1058.5	1376	1.389	0.39564	3.510767364
ndhH-1	36	26	503	965	2.7435	0.66528	4.123827561
ndhH-2	36	33	438	803.5	2.718	0.95928	2.833375031
rps15	36	33	412	832	2.064	0.93744	2.201740911
rps15-IG	36	27	892	1676	3.989	0.96096	4.151057276
ycf1-1	36	36	147	346	0.713	0.28056	2.54134588
ycf1-2	36	36	136.5	547	0.555	0.29568	1.877029221
ycf1-3	36	33	211.5	365	1.848	0.67872	2.722772277
ycf1-4	33	30	103.5	371	2.451	1.12812	2.172641208
ycf1-5	35	30	3516.5	9785	2.7	1.27932	2.110496201

ycf1-6	36	31	2938	28890.5	3.075	1.18104	2.603637472
ycf1-7	36	36	155.5	365	1.1995	0.60396	1.986058679
ycf1-8	36	31	142	330.5	1.1665	0.59808	1.950407972
ycf1-9	36	36	94	161.5	0.8695	0.4746	1.832069111
N-GUU-2	36	36	98.5	239	0.1785	0.3066	0.582191781
IG N-R	36	36	474	705.5	1.072	0.73584	1.456838443

[1] each probe is spotted with 12 replications per chip; spots with less than 5 detectable spots were removed from the data set

[2] differential enrichment of IP over control- IP, based on the median of ratios of red (635 nm) or green (532 nm) fluorescence

Supplemental Table 2: Oligo RIP-Chip data for Figure 3b and Figure S1

Name	sequence of oligonucleotide	length of probe (nt)	start of probe[1]	end of probe[1]	Spot Count CP33B [2]	Spot Count control [2]	Median(F635 Median - B635) CP33B [3]	Median(F635 Median - B635) control [3]	Median(F532 Median - B532) CP33B [3]	Median(F532 Median - B532) control [3]	Median(Median of Ratios (635/532)) CP33B [4]	Median(Median of Ratios (635/532)) control [4]	Diff. Enrich. [5]
psbA-1	taggaatattttttccaaatcatatgaatacgattgaatacgatccgtag	50	1667	1716	48	27	68	9	79.5	90	1.27	0.20	6.53
psbA-2	atagccatgtcaaccaatgtaaaatggataagatccttttagtttagatt	50	1547	1596	36	33	59.5	14	89.5	85	0.86	0.28	3.13
psbA-3	gagcttgttatgaaacagtataacatgacttatatagccatgtcaaccaa	50	1514	1563	48	33	111	25	96	94	1.69	0.20	8.31
PsbA-4	gcgcacaaattctctaagtagataattgagagcttgttatgaaacagtat	50	1485	1534	48	30	40394	1313.5	7315.5	25633.5	5.64	0.06	90.95
psbA-5	tttatttaataatcagggtataactcccaagcgcacaaattctctaagta	50	1459	1504	48	33	29363	1413	7950.5	27299	4.85	0.06	86.57
psbA-6	ctaaaattgcagtcatggtaaaatccttggtttatttaataatcagggta	50	1429	1476	48	33	28292.5	2104	5956	28785	6.53	0.07	98.98
psbA-7	aagcgaccccataggctttcgctttcgcgtctctctaaaattgcagtcat	50	1395	1444	48	30	44462.5	2041	6804.5	35430.5	7.46	0.06	124.41
psbA-8	taaggtagggatcatcaaaacaccaaaccatccaatgtaaagacggtttt	50	1322	1371	48	33	39141.5	1153	5379	19246	6.86	0.07	102.40
psbA-8a	actggaggagcagcaatgaatgcgataataaaaacagaagttgcggtcaa	50	1272	1321	24	12	14389.5	1325.5	5979	15505	2.91	0.09	33.25
psbA-9	cacgaataccatcaatatctactggaggagcagcaatgaatgcgataata	50	1252	1301	48	33	32427.5	2353	7123	30672	4.81	0.10	50.11
psbA-9a	ggcaccggaaataatattgtttccgtaaagaagagatccagaaacaggtt	50	1202	1251	24	12	7463.5	1546	7606	18948	1.89	0.08	22.69
psbA-9b	tcccagattgggtaaaaatgcaatccaatagctgcagaagtaggaataat	50	1152	1201	24	12	19085	1642	6884.5	21050	3.87	0.08	47.25
psbA-9c	ttagttcataaggaccgccgttgtatagccattcatcaacggatgcagct	50	1102	1151	24	12	11171	2530.5	7021	27748.5	7.14	0.09	75.93
psbA-9d	ccactcacgacccatataacaagctacaccaagtaaaaagtgtagaacaa	50	1052	1101	24	12	24155.5	2220.5	10961.5	25620	4.43	0.09	49.45
psbA-10	aggacgcatacccagacggaaactaagttcccactcacgacccatataac	50	1022	1071	48	30	30714	1757	3589	28843	12.85	0.07	191.86
psbA-10a	aaaacagcagtcgcagctgcaacaggagctgaatatgcaacagcaatcca	50	972	1021	24	12	9143	1447.5	4228	16924	3.02	0.09	33.88
psbA-10b	ctagaggcataccatcagaaaaacttccctgaccaattggatagatcaag	50	922	971	24	12	25687	2615.5	7574	26056.5	4.73	0.10	47.58
psbA-10c	gttgtgctcagcctggaatacaatcataaagttgaaagtaccagagattc	50	872	921	24	12	16756.5	1650.5	6521	20020	5.80	0.08	70.67
psbA-10d	ccgccgaatacaccagctacacctaacatgtgaaatgggtgcataagaat	50	822	871	24	12	21139.5	1975	6143	23275	5.58	0.09	64.11
psbA-10e	tgatcaaactagaagttaccaaggaaccatgcatagcactaaaaagggag	50	772	821	24	12	17021.5	2277	6088.5	23926.5	4.66	0.10	45.94
psbA-11	cattagcagattcattttctgtggtttccctgatcaaactagaagttacc	50	742	791	48	33	29557.5	1105	5638.5	21516	6.57	0.06	111.32
psbA-11a	agcagctacaatgttgtaagtttcttcttcttgcccgaatctgtaacctt	50	692	741	24	12	35475	5512.5	8766	53834.5	7.66	0.11	68.38
psbA-11b	gaattgttgaaactagcatattggaaaatcaatcggccaaaataaccgtg	50	642	691	24	12	5377	2866	3421.5	29905.5	13.38	0.10	140.81
psbA-11c	accaaatacctactaccggccaagccgctaagaagaaatgtaaagaacga	50	592	641	24	12	33079.5	3175	3191	31380	16.73	0.10	166.50
psbA-11d	attgaaaccatttaggttgaaagccatagtactaatacctaaagcagtaa	50	542	591	24	12	27589	3315.5	3255.5	34282.5	11.23	0.10	111.14
psbA-12	tccttgactatcaactactgattggttgaaattgaaaccatttaggttga	50	512	561	48	33	27935.5	1120	2757.5	19581	12.11	0.06	201.89
psbA-12a	cggttaataatatcagcccaagtattaataacacgtccttgactatcaac	50	477	526	24	12	30931.5	3680.5	3929.5	40838	18.35	0.09	199.47
psbA-13	tacgttcatgcataacttccataccaaggttagcacggttaataatatca	50	442	491	48	30	46157	1372.5	2605	32042	13.87	0.06	247.68
psbA-13a	aacagcagctaggtctagagggaagttgtgagcattacgttcatgcataa407	53	407	456	24	12	7107.5	1765	3836.5	19696	13.95	0.09	156.70

psbA-14	acactaacgaattatccatttgtagatggagcctcaacagcagctaggtc	50	372	421	48	33	32304.5	1594	2694.5	26739	13.85	0.06	216.40
psbA-15	aagaaggcttatattgctcgtttttttactaaactagatctagactaacac	50	326	375	48	30	26378	490.5	3812.5	12585	5.88	0.06	105.92
psbA-16	acgagtaatataagccctctttcttatttaaagaaggcttatattgctcg	50	296	345	48	33	670.5	32	203	461	5.31	0.07	71.76
psbA-17	ataaaaatttgctcatttttatagaaaaaaacgagtaatataagccctct	50	266	315	48	33	1204.5	48	310.5	730	4.40	0.06	71.02
psbA-17a	ttattattatttattattaatataataaataaagtaaaatatgatactct	50	216	265	23	12	697	27	141	414	4.92	0.08	58.92
psbA-18a	tagcaaattccaccttatttttttttctaataaaaaatatatagtaatttt	50	156	205	23	12	89	20	120	281	0.81	0.09	8.71
psbA-19	tgaacccgcgatggtgaattcacaatccactgccttaatccacttggcta	50	20	69	48	33	1420.5	1570	31671	27725	0.07	0.06	1.16
rps14	ttttccaatttttgcctcttcttctccctataaatcaaacttttctttgc	50	37188	37237	48	30	517	66.5	884	777.5	0.883	0.0685	12.89051095
rsp14-psaB	aataaatataagagtgcatacctatatttttttttattaaaaaaaatatat	50	37300	37349	48	30	120	18.5	174	153.5	0.6735	0.1215	5.543209877
psaB1	ttaaccgaatttgcccgatgtggaggcaatcaagaaagccgcataagtga	50	37375	37424	48	30	226.5	38.5	408	530.5	0.7795	0.0785	9.929936306
psaB2	atatccattgataagttgtgaagagtttaaccatagataatctcttaacc	50	37675	37724	48	30	484.5	73	959.5	892.5	0.6185	0.0815	7.588957055
psaB3	tactaagatcaatgtagttgtatgtaaacctaaagcaatagcatgatgaa	50	37975	38024	48	30	408.5	67	731.5	798	0.786	0.0795	9.886792453
psaB4	tcccaaagtatggaaccctagaaagaggctggcccaacttaaatgggata	50	38275	38324	48	30	124	27.5	254.5	287	0.6015	0.1045	5.755980861
psaB5	gcctaattgaaaatgaatcgaattattgattgtgtcataaagacccttat	50	38575	38624	48	30	402	64.5	759.5	964	0.5465	0.068	8.036764706
psaB6	ttgagcataaagattccactgacccgtaaaaagtgggcctaacccttggg	50	38875	38924	48	29	343.5	59	767.5	856	0.5465	0.07	7.807142857
psaB7	aagatcttcattagtacgtaaaccgattgtataccaccactgataaacac	50	39175	39224	48	30	404.5	60	763	802.5	0.739	0.08	9.2375
psaB8	atcatgactctcgaagtcatgtgcggtagcaataccaaaccaaatacgac	50	39475	39524	48	30	272.5	45	378.5	487	0.8335	0.092	9.059782609
psaB-psaA	ttggctaaaccttggaaatcttaatgccataatgcctttcaaatcctcc	49	39550	39598	48	30	484.5	72.5	694	744	0.872	0.0725	12.02758621
psaA1	tagccattatcctactgcaataattcttgctaagaagaacgcccatgtt	49	39605	39647	48	30	422.5	69.5	723.5	742	0.7775	0.0775	10.03225806
psaA2	tgcccataagaaatcgcggagccacccattaatagtaatggaactctgtg	50	39905	39954	36	24	311.5	53.5	416	467.5	0.753	0.124	6.072580645
psaA3	acctttcaacagtatcaataccgtcacatgaattgtaaatgcatgaatat	50	40205	40254	48	36	505	75.5	867	692	0.7415	0.0945	7.846560847
psaA4	accaaaactgtggaagcctagaaatatacatacccagttgagatgtgata	50	40505	40554	48	36	341	60	640.5	484.5	0.828	0.104	7.961538462
psaA5	agataattgagcatgccatgatgttgttagaatttcatataggcctttat	50	40805	40854	48	34	299.5	49	629	469	0.5715	0.0955	5.984293194
psaA6	ataaagttgagccaaaagatcccgattcaagataaattcatgaggaagcg	50	41105	41154	48	33	177.5	41	368.5	294	0.5935	0.095	6.247368421
psaA7	agttattccggatgctcgccaaatctgaaaaaagcctgaggttatttgta	50	41405	41454	48	33	211.5	39	412	396	0.626	0.117	5.35042735
psaA8	agcatgtaggttccagatccaagtggtagtatcaggtcccttagctattg	50	41705	41754	48	33	393	65	693.5	717	0.6375	0.09	7.083333333
psaA-IG	gaataatcattgagtcctcctctttccggacaacacatacaaagaaacc	49	41848	41896	48	33	315.5	61	749	678	0.596	0.089	6.696629213
psaA-410	attatcctatttcaataaatgcttattagtcattactaagaaaaagtcta	50	42217	42266	40	28	49	17	63.5	57.5	0.7545	0.4345	1.736478711
ndhB-1	aaaaaaaccaatttttgatttttggaatggaatatttacggaatccccat	50	97241	97192	48	33	71.5	21	323	249	0.362	0.089	4.06741573
ndhB-2	attctcgtagaatcgagaatgaaattttcattctgtacatgccagatcat	50	97164	97115	48	33	73.5	18	247.5	187	0.442	0.158	2.797468354
ndhB-3	atcggaacaatagggccgttatgctcattacgaaacttgttgaagagatg	50	96979	96930	48	32	83.5	20	251	170	0.5355	0.147	3.642857143
ndhB-4	ttcctcctagagtagctgttaatatgaataacagaaactctgttatagcc	50	96793	96744	48	33	66.5	26	238	186	0.3645	0.122	2.987704918
ndhB-5	tagccaagagaaaccatgaaccagaatagaagagcttgccccacccatga	50	96611	96562	48	30	94	19.5	332	326.5	0.323	0.0845	3.822485207
ndhB-6	tccttcgtatacgtcaggagtccattgatgagaaggggctagggaaagct	50	96437	96388	48	30	31.5	10.5	117	94	0.425	0.1775	2.394366197
ndhB-7	gaaaagcaacgactggagtgggagatccttcgtatacgtcaggagtccat				48	30	24	9.5	96	86	0.3815	0.2065	1.847457627

ndhB-8	tagagaggtaggaatttctcaaacgaaccgcactccttcgtatacgtcag	50	96404	96355	45	30	32	11	124	92.5	0.393	0.199	1.974874372
ndhB-9	gcatgtccatagagttttgaaaaatccaaacatctcagagatagatagag	50	96359	96310	44	30	27	13.5	116.5	90	0.5025	0.1685	2.982195846
ndhB-10	gtcaaagttctgtcttggtctgcgtggggatagcatttctcttctgcatg	50	96314	96265	48	30	82.5	16.5	127	104.5	0.6805	0.162	4.200617284
ndhB-11	ttcgttgttcctgaccctgcttcaccttaattgttatttgaacaagtcaa	50	96269	96220	48	30	49	11.5	160	143.5	0.495	0.1195	4.142259414
ndhB-12	ccgtaataacaaacttgcaaaatggatccgtttatcataaagagattcgt	50	96224	96175	46	30	35	12.5	119	86.5	0.674	0.2225	3.029213483
ndhB-13	gtctttcttgtatacgtcattagtccgatcctttgcaggaactacccgta	50	96179	96130	47	30	49	16.5	139	129	0.542	0.132	4.106060606
ndhB-14	tgaaggatgagaaccaactatgtagcatctacatcgagaattcaagtctt	50	96134	96085	47	30	47	17.5	139	152	0.54	0.154	3.506493506
ndhB-15	ggtgatccttttgtcgacggatgctcctattacactcgtagtctctgaag	50	96089	96040	47	30	33	15.5	132	122.5	0.469	0.1415	3.314487633
ndhB-16	atctgatttgattcgttctcaatagccatgagattatcatcttagggtga	50	96044	95995	48	30	45.5	16	157	139	0.4995	0.1405	3.555160142
ndhB-17	ccttggttccgtaggagcacgtccgaaagattgagaaatggaaccatctg	50	95999	95950	48	33	63.5	33	193	141	0.4605	0.442	1.041855204
ndhB-18	cttcatcagtggttgtgaatgactgatttttctcaatcttttcgaccttg	50	95954	95905	48	33	46.5	17	132	113	0.4905	0.154	3.185064935
ndhB-19	tgtataagatcgaatcctttctatttctaaaacggattactaatccttaa	50	95890	95841	48	33	30.5	15	105	85	0.4795	0.219	2.189497717
ndhB-20	agtgataaaagtaaagaagaactcatcttctttctttttgattactttc	50	95832	95783	39	33	40	8	119	82	0.34	0.206	1.650485437
ndhB-21	caaaaccgtgcatgagactttcatctcgcacggctcctaagtgataaaag	50	95793	95744	48	33	28.5	10	98	88	0.4345	0.254	1.710629921
ndhB-22	gtcagagtcgaaaagaggattcctcacttctttctctcattcaaaaccgt	50	95752	95703	48	33	38	17	134.5	129	0.497	0.155	3.206451613
ndhB-23	tccgatttgacctatggacgaatatgcaagcatacgtttcatgcttgttt	50	95530	95481	48	33	92.5	29	350	341	0.3725	0.09	4.138888889
ndhB-24	gagctaaagagagagccaaaaaaggatcttttgtgtataatcctgcataa	50	95331	95282	48	33	49.5	15	157	118	0.4725	0.156	3.028846154
ndhB-25	taataacttgattatttttagataatagtagatagaaagaacgctcgtaa	50	95167	95118	47	33	54	22	220	186	0.439	0.134	3.276119403
ndhB-26	ctagaagctaaaaagggtatcctgagcaatcgcaataatcgggttcattg	50	94990	94941	48	33	49.5	15	171	116	0.4785	0.205	2.334146341
ndhB-27	cattttgggcggaaccgatctactaattctttgattccagttagtattag	50	94910	94861	48	33	59.5	25	221.5	218	0.425	0.132	3.21969697
ndhF-20	ctcgaagtttccttcgtaacccccggaacttcttcgtagtggctccgttc	50	109146	109195	48	30	143	67.5	1144.5	849	0.1665	0.07	2.378571429
ndhF-19	tgtgatggtttttcaatcttttatactaggtaatctagtatccttatgca	50	109404	109453	48	30	38.5	16.5	187.5	136.5	0.3375	0.129	2.61627907
ndhF-18	accacattttccatagggccctcttatctcttccttctccgagctcgggt	50	109504	109553	45	24	19	16	95	75	0.315	0.2145	1.468531469
ndhF-17	gttttattgcgggacagctcatgatgttcatatcgatctattatgcgcct	50	109604	109653	48	30	29	13	173	133	0.307	0.1235	2.48582996
ndhF-16	gtatcttttgtttcatttcttctggaacaatcacaaacactttttgatt	50	109704	109753	48	30	38.5	17	164	139	0.313	0.1365	2.293040293
ndhF-15	ttcctgaataatctcatttttcaattattcaaccatttcattttaccaag	50	109804	109853	48	30	34.5	18.5	135	111	0.3615	0.178	2.030898876
ndhF-VL	agtcaacatttatatgtttcgatgcaacaacaagatgttatttgtaacaa	50	109872	109921	48	30	31.5	11	186	99.5	0.3505	0.178	1.969101124
ndhF-14	agatgttatttgtaacaagtagttttgttggttggttaattggtcacatt	49	109904	109953	48	33	107.5	25	515.5	258	0.434	0.106	4.094339623
ndhF-mature	gtagttttgttggttggttaattggtcacattttattcatgaaatgggtt	50	109922	109971	48	30	72	16	298.5	150.5	0.341	0.142	2.401408451
ndhF-13	gcaaaataattctattaggtctaatgtagttattagatctaataagtat	49	109995	110044	48	33	53	22	170	126	0.409	0.172	2.377906977
ndhF-12	ctcttatttattacctgtgtctactatttaggcagaataccatcacccat	50	110095	110144	48	33	110.5	29	478.5	471	0.2815	0.1	2.815
ndhF-11	accaagaggtatccaccgaagaagctccttttccttctctttttttcggaa	50	110195	110244	48	32	300.5	151.5	3364.5	3193	0.1515	0.0555	2.72972973
ndhF-10	tggaaaagacaaaattaataaggatgatgaattccacgttcgaacatact	50	110295	110344	48	33	67.5	24	462.5	344	0.285	0.077	3.701298701
ndhF-9	aagaaaattcgaatttagaatttttcaaaataaaaaaaaaagaggatcat	50	110384	110433	36	24	26	9	63.5	54.5	0.5025	0.3325	1.511278195
ndhF-8	aattaagtaaggttaaatttaataaagatgaataaaaaggcttatataaa	50	110724	110773	48	33	35	10	139.5	85	0.531	0.218	2.435779817

ndhF-7	tttttattaagttttttttcttcttctttaccccataaagagagtgaata	50	111170	111219	47	32	36	11.5	124	81.5	0.5	0.2405	2.079002079
ndhF-6	gatcctaaaaacaacaaagctttcgaataagcatgagtaatcaaatgaa	49	111560	111609	48	33	54	14	251.5	127	0.4155	0.152	2.733552632
ndhF-5	atagaaatgcacacaaagtaaggaataagagatttactctattatttaaa	50	111942	111991	48	33	44	10	207	94	0.4255	0.191	2.227748691
ndhF-4	acatataattgtcactataaatcagaaccaaaatcccaacagttgtaatt	50	112293	112342	46	33	34	13	137.5	61	0.5525	0.294	1.879251701
ndhF-3	tgatccatgaatattgatatgtatgttccataaaataaaaaatccttttt	50	112608	112657	43	33	26	11	95	77	0.433	0.286	1.513986014
ndhF-2	ttgtgattttatgcaaatacagaaaaagtgaattataattccatattaca	50	112909	112958	48	30	18	7	85	66	0.3905	0.234	1.668803419
psbD-prom	ctttttttgcgtgtattaaatacttgtctattaaatactatagtatcaact	50			48	30	475	26.5	747.5	640.5	0.723	0.0565	12.79646018
psbD-IG	tggtaaatttaccaagggctatagtcattagcgatcctcctattc	45			48	30	694	52	1113.5	935.5	0.7625	0.0685	11.13138686
psbD2	gcaccatgaatagcgcatagcagagccgcgcccagtacaccggcgactcc	50	121119	121168	48	30	305.5	46	688	703.5	0.762	0.073	10.43835616
psbD3	gtatagaaagtctcaaattctggatcttccgctgcacggatttcctggga	50	120819	120868	48	30	1347	115	2532	2231.5	0.769	0.072	10.68055556
psbD-psbC	agctaaagttaaagagcgtttccacgtggtagaacctcctcagggaatat	50			48	30	749	83	1106.5	1038.5	0.7875	0.081	9.722222222
psaJ	aatgcatctggaaataaacgattaatctctattaataaacctgctaacga	50	87441	87490	48	33	338.5	90	4413.5	2386	0.134	0.072	1.861111111
trnF	atttgaactggtgacacgaggattttcagtcctctgctctaccaactgag	50	106245	106294	48	33	245	868	15360	18463	0.041	0.066	0.621212121
trnT					48	30	234	294.5	6785	5507	0.0545	0.068	0.801470588
trnL(CAA)_s					48	30	537	991	20361.5	22864	0.039	0.044	0.886363636

[1] oligo nucleotide genome position in NC_000932.1

[2] each probe is spotted with 12 replications per chip; spots with less than 5 detectable spots were removed from the data set

[3] the median of red (635 nm) or green (532 nm) fluorescence, from the replicates of each probe is calculated, including a local background substraction, performed by the GenePix Pro 6.0

[4] median of ratios of the background red (635 nm) and green (532 nm) fluorescence

[5] differential enrichment of IP over control- IP, based on the median of ratios of red (635 nm) or green (532 nm) fluorescence

Supplemental Table S3: summary of sites in the chloroplast genome of Arabidopsis that correspond to the top motif of the RBNS analysis.

	target sites		
RBNS motif [1]	*psbA* CDS[2]	*psbA;* non-translated[2;3]	other [No]
GGUUACU	/	/	16
GGCUAUU	/	/	24
GGCUACU	/	/	10
GGUUAUU	688-682	/	40

[1] The RBNS top motif was further separated into four distinct sequences based on C/U ambiguities at position 3 and 6.

[2] target site position within the Arabidopsis thaliana chloroplast genome (NC_000932.1)

[3] target sites in the intergenic regions between *trnK* and *psbA* and between *trnH* and *psbA.*

Supplemental Table 4: RIP-chip data for Fig. 5B (CP33B RIP-chip from membranes)

Name			Median(F532 Median - B532)		Median (Median of Ratios (635/532))		Diff. Enrich. [2]
	Spot Count CP33B [1]	Spot Count control [1]	CP33B	control	CP33B	control	(CP33B/ control)
psbA	12	12	4457.5	5758	2.3667	0.068	34.80441176
psbA-Kex2	12	12	864	979.5	0.9863	0.13	7.586923077
Kex2-matK	12	12	836.5	628.5	0.5026	0.277	1.814440433
matK-1	12	12	1170.5	648.5	0.5852	0.3405	1.718649046
matK-2	12	12	1643.5	1407.5	1.0892	0.4695	2.319914803
Kint	12	12	1036.5	670.5	0.8155	0.3675	2.219047619
Kint-Kex1	12	12	597.5	506	0.6818	0.3815	1.787155963
IG_K-rps16	12	12	1451.5	799.5	0.8372	0.4115	2.034507898
rps16ex2	12	12	1752.5	1322	1.1431	0.4855	2.354479918
rps16ex2-in-ex1	12	12	1044.5	761	0.6769	0.397	1.705037783
rps16in-1	12	12	1048.5	697	0.9583	0.4455	2.151066218
rps16in-2	12	12	712.5	474	0.8218	0.412	1.994660194
rps16ex2-IG	12	12	1419	1344.5	0.7427	0.4125	1.800484848
Q	12	12	7077	2449	0.1526	0.089	1.714606742
psbI-S	12	12	1477	1394	0.4249	0.1765	2.407365439
IG-psbI-G-1	12	12	16009.5	14259	0.0546	0.031	1.761290323
IG-psbI-G-2	12	12	2110	1676.5	0.3409	0.152	2.242763158
Gex1-in	12	12	5869	3894	0.3486	0.098	3.557142857
in-Gex2	12	12	3558	2900	0.3059	0.117	2.614529915
Gex2-R	12	12	2145.5	1063	0.7014	0.2405	2.916424116
atpA-1	12	12	1168	840.5	0.9646	0.4375	2.2048
atpA-3	12	12	573	383	0.8008	0.296	2.705405405
atpA-atpFex2	12	12	645.5	646	0.8015	0.284	2.822183099
atpFex2-in	11	12	1548	1205	1.0262	0.2915	3.520411664
atpFex2-in-ex1	12	12	2410	2201.5	0.707	0.2725	2.594495413
atpFin-ex1	12	12	989	782	0.7371	0.2995	2.461101836
atpFex1-IG	11	12	1902	2310.5	0.8652	0.272	3.180882353
atpH	12	12	882.5	743.5	0.8113	0.3215	2.52348367
atpH-IG	12	12	6638	8399	1.1746	0.229	5.129257642
IG-atpI	12	12	1084.5	1240.5	0.7973	0.339	2.351917404
atpI	12	12	1248	954	0.8526	0.3045	2.8
atpI-rps2	12	12	2089	2330	0.8645	0.3	2.881666667
rps2	12	12	1651	1088.5	0.7665	0.38	2.017105263
rps2-IG	11	12	859	768.5	0.5698	0.377	1.511405836
rpoC2-1	12	12	1239.5	1036	0.6958	0.36	1.932777778
rpoC2-2	10	12	1298	810	0.5502	0.352	1.563068182
rpoC2-3	12	9	1650.5	274	0.5761	0.396	1.45479798
rpoC2-4	12	12	1585	980	0.5131	0.3695	1.388633288
rpoC2-5	12	12	1038	804	0.56	0.3745	1.495327103
rpoC2-6	12	12	1752.5	1123	0.511	0.3445	1.483309144

rpoC2-rpoC1-2	12	12	820	621.5	0.5082	0.3835	1.325162973
rpoC1ex1-1	12	12	1645	1042.5	0.5278	0.362	1.45801105
rpoC1ex1-2	12	12	1093.5	833.5	0.4179	0.3325	1.256842105
rpoC1ex1-in	12	12	873	564.5	0.5803	0.3625	1.600827586
rpoC1ex1-in-ex2	12	12	1454	937.5	0.5397	0.369	1.462601626
rpoC1in	12	12	1294.5	885.5	0.518	0.3615	1.432918396
rpoC1in-ex2	12	12	1348.5	991.5	0.6062	0.4045	1.498640297
rpoC1-rpoB-1	12	12	1960.5	1337	0.5579	0.4245	1.314252061
rpoC1-rpoB-2	12	12	2637.5	1645	0.5992	0.371	1.61509434
rpoB-1	12	12	1306	887	0.3346	0.315	1.062222222
rpoB-2	12	12	454	467.5	0.5264	0.3355	1.56900149
rpoB-3	12	12	1925	1180	0.8379	0.4415	1.897848245
rpoB-4	12	12	1035	577.5	0.5558	0.429	1.295571096
rpoB-IG	12	12	1195	952.5	0.6741	0.4415	1.526840317
IG rpoB-C	12	12	1253.5	1271	0.6566	0.4295	1.528754366
IG-C	12	12	4520	3696	0.1813	0.095	1.908421053
C-IG	12	12	374	383	0.4508	0.3775	1.194172185
IG-petN	12	12	1517.5	1083	0.4802	0.257	1.86848249
petN-IG	12	12	255.5	279.5	0.5782	0.325	1.779076923
psbM	12	12	477.5	309	0.63	0.3565	1.767180926
IG psbM-D-1	12	12	829.5	574.5	0.5537	0.339	1.633333333
IG psbM-D-2	12	12	1017.5	541.5	0.7224	0.4405	1.639954597
D	12	12	1094.5	1262.5	0.2814	0.1625	1.731692308
Y	12	12	644	436	0.6489	0.3145	2.06327504
E	12	12	1839	1423	0.7644	0.344	2.222093023
IG E-T	12	12	1399.5	923.5	0.8162	0.4505	1.811764706
T	12	12	941.5	982.5	0.5201	0.246	2.114227642
IG T-psbD-1	12	11	1835.5	1808	0.6405	0.289	2.216262976
IG T-psbD-2	12	12	2834	1580.5	0.9905	0.264	3.751893939
psbD-1	12	12	3610.5	2070.5	1.6485	0.354	4.656779661
psbD-2	12	11	1242	1198	0.6895	0.228	3.024122807
psbD-psbC	12	12	767.5	807	0.7882	0.2515	3.133996024
psbC-1	12	12	1182.5	1291.5	0.4648	0.168	2.766666667
psbC-2	12	12	2474.5	2403.5	0.7833	0.171	4.580701754
psbC-S	12	12	2126	2261.5	0.6433	0.2055	3.130413625
S-psbZ	12	12	2275	2184	0.9324	0.311	2.99807074
IG psbZ-G	12	12	1567.5	1228.5	0.9597	0.343	2.797959184
G-fM	12	12	1072.5	933.5	0.2912	0.1405	2.072597865
fm-rps14	12	12	2645.5	2093	0.182	0.096	1.895833333
rps14-psaB-1	12	12	3391	2760.5	0.5684	0.3125	1.81888
rps14-psaB-2	12	12	2662.5	2047	0.6741	0.334	2.018263473
psaB-1	12	12	5786	5122	1.3097	0.403	3.249875931
psaB-2	12	12	1296.5	646	1.1025	0.415	2.656626506
psaB-psaA-1	12	11	1230	1080	0.9373	0.372	2.519623656
psaB-psaA-2	12	12	1140	1095	0.8995	0.394	2.282994924
psaA-1	12	12	4568	3143	0.8127	0.3335	2.436881559
psaA-2	12	12	1422.5	1184	1.2012	0.4545	2.64290429
psaA-3	12	12	4696.5	3557	1.8214	0.439	4.148974943

psaA-IG-1	12	12	4148	2448	1.1802	0.363	3.251239669
psaA-IG-2	12	12	863	601.5	1.1382	0.4465	2.549160134
IG-ycf3ex3	12	12	1440	1223.5	0.9758	0.437	2.232951945
ycf3ex3-in2	12	12	1342.5	1030.5	0.7966	0.4235	1.880991736
ycf3ex3-in2-ex2	11	11	1004	720	0.6188	0.396	1.562626263
ycf3in2	11	12	915	605	0.8386	0.424	1.977830189
ycf3ex2	12	12	638.5	470	0.6433	0.4175	1.540838323
ycf3in1	12	11	2184.5	1132	0.9436	0.438	2.1543379
acf3ex1	12	12	1717	1111	0.7903	0.435	1.816781609
S-GGA	12	12	4125	4148	0.1365	0.0765	1.784313725
rps4-1	12	12	1270	968	0.8183	0.4555	1.796487377
rps4-2	12	12	866.5	648.5	0.5187	0.4085	1.269767442
T-UGU	12	12	1016.5	510	0.5201	0.3385	1.53648449
T-IG	12	12	3035.5	2104	0.1547	0.088	1.757954545
L-UAA ex1-in	12	12	5904	4140.5	0.1029	0.074	1.390540541
L-UAA in-ex2	12	12	2197	2892	0.175	0.1005	1.741293532
L-UAA ex2-IG	12	12	4135	3661	0.3661	0.1985	1.844332494
F-GAA-1	12	12	3121	2173.5	0.07	0.065	1.076923077
F-GAA-2	12	12	1695	913.5	0.5481	0.285	1.923157895
ndhJ	12	12	1105.5	1152	0.6209	0.3225	1.925271318
ndhJ-ndhK	12	12	1400.5	1040.5	0.595	0.4015	1.481942715
ndhK	12	12	699	557	0.7931	0.4025	1.970434783
ndhK-ndhC	12	12	1356	873.5	0.8785	0.376	2.33643617
ndhC-IG	12	12	3661.5	3052.5	1.1648	0.395	2.948860759
IG ndhC-V-UAA	12	12	1643	1249.5	0.8967	0.415	2.160722892
Vex2-in	12	12	1222	824	0.7315	0.396	1.847222222
Vin-ex1	12	12	2030.5	1418	0.3297	0.234	1.408974359
Vex1-atpE	12	12	3249	3188	0.1946	0.1035	1.880193237
atpE-atpB	12	12	1130	1177.5	0.4655	0.3085	1.5089141
atpB-1	12	12	2606	3574.5	0.5138	0.306	1.679084967
atpB-2	12	12	1508	1120	0.7651	0.3345	2.287294469
atpB-3	12	12	2131	1702	0.581	0.303	1.917491749
atpB-rbcL	12	12	2616.5	2865	0.406	0.2395	1.69519833
rbcL-1	12	12	9203.5	6701	0.2282	0.0845	2.700591716
rbcL-2	12	12	1573.5	1632	0.1148	0.0665	1.726315789
rbcL-3	12	12	7827	7955.5	0.1883	0.0635	2.965354331
IG rbcL-accD	12	12	1902.5	1802	0.5362	0.2945	1.820713073
accD-1	12	12	1004	668	0.5684	0.2965	1.91703204
accD-2	12	12	1818	1436	0.4893	0.2795	1.750626118
accD-3	12	12	1365.5	1240.5	0.7315	0.3735	1.958500669
IG accD-psaI	12	12	2184	1587	0.7203	0.3925	1.835159236
psaI	12	12	1399.5	845	0.5285	0.327	1.616207951
ycf4-1	12	12	2885.5	2116.5	0.5334	0.274	1.946715328
ycf4-2	12	12	1570	1324	0.6223	0.318	1.956918239
cemA-1	11	12	1437	1559	1.085	0.418	2.59569378
cemA-2	12	12	2138.5	1641	0.9191	0.3605	2.549514563
cemA-3	12	12	2536	1587.5	0.7322	0.3145	2.328139905
petA-1	12	12	1126	905	0.5775	0.3175	1.818897638

petA-2	12	12	2548.5	2152.5	0.5432	0.2545	2.134381139
petA-3	12	12	1683.5	1161.5	0.875	0.4045	2.1631644
IG petA-psbJ	12	12	1937.5	1518	0.5705	0.2725	2.093577982
psbJLF	12	12	6727.5	3590	0.1281	0.1045	1.225837321
psbFE	12	12	3162	2952	0.3794	0.1695	2.238348083
IG psbE-petL-1	12	12	3746.5	1869	0.5061	0.209	2.4215311
IG psbE-petL-2	12	12	1262	772.5	0.7182	0.357	2.011764706
IG psbE-petL-3	12	12	1510.5	627.5	0.7903	0.3615	2.186168741
petL	12	12	3732.5	2709	0.4739	0.2335	2.029550321
petG-W-CCA	12	12	2216.5	1493	0.5698	0.258	2.208527132
P-UGG	12	12	2695.5	2575	0.2765	0.106	2.608490566
psaJ	12	12	5939.5	4665	0.175	0.083	2.108433735
IG psaJ-rpl33	12	12	3247	3049	0.7602	0.279	2.724731183
rpl33	12	12	2269.5	1008.5	1.0598	0.325	3.260923077
rpl33-rps18	12	12	4177.5	3253	0.9191	0.345	2.664057971
rps18	12	12	1816.5	1466.5	0.8316	0.375	2.2176
rpl20	12	12	1455	975	0.63	0.405	1.555555556
rpl20-IG	12	12	1363.5	776.5	0.6783	0.4555	1.489132821
in1-rps12ex1	12	12	1374.5	1564	0.5978	0.364	1.642307692
rps12ex1-clpPex3	12	12	2239	1635.5	0.665	0.299	2.224080268
clpPex3-in2	12	12	2380	1408.5	0.6993	0.3825	1.828235294
in2-clpPex2	12	12	1650.5	1472.5	0.5992	0.388	1.544329897
clpPex2-in1	12	12	1859	770.5	0.6713	0.365	1.839178082
clpPin1	12	12	2228.5	1900.5	0.5663	0.3075	1.841626016
clpPex1	12	12	4715.5	3739.5	0.7588	0.2365	3.20845666
psbB-1	12	12	7451	5125.5	0.4557	0.168	2.7125
psbB-2	12	12	1763.5	1683	0.3297	0.169	1.950887574
psbBTN	11	12	1216	1839	0.686	0.2385	2.876310273
psbNH	12	12	2911	2097	0.2695	0.114	2.364035088
psbH-petBex1-in	12	12	2030.5	1380	0.5859	0.2985	1.96281407
petBin	12	12	2083.5	1582	1.1102	0.433	2.563972286
petBin-ex2	12	12	5248.5	3805	0.8302	0.195	4.257435897
petBex2	12	12	2270	2036.5	0.3472	0.1615	2.149845201
petBex2-petDex1-in	12	12	2784.5	2435.5	0.5187	0.2015	2.574193548
petDin	12	12	2353.5	1589	0.6314	0.279	2.263082437
petDex2	12	12	2714	2913.5	0.7511	0.189	3.974074074
petD-rpoA	12	12	1002	915	0.5544	0.316	1.75443038
rpoA	11	12	2050	1491.5	0.693	0.3655	1.896032832
rpoA-rps11	12	12	1770	1037.5	0.6503	0.3785	1.718097754
rps11-rpl36	12	12	1898.5	794.5	0.6657	0.3735	1.782329317
IG rpl36-rps8	12	12	977	1226	0.7455	0.3725	2.001342282
rps8	12	12	1092	1409	0.7987	0.4425	1.804971751
rps8-rpl14	12	12	2448.5	1729	0.8554	0.386	2.216062176
rpl14	12	12	1683.5	1318	0.7007	0.336	2.085416667
rpl16ex2	12	12	1151.5	790.5	0.7007	0.4005	1.749563046
rpl16in-1	12	12	3242	2742.5	0.9037	0.4155	2.174969916

rpl16in-2	12	12	825	440	0.8316	0.423	1.965957447
rpl16in-ex1	12	12	1895	1703	0.8323	0.4475	1.859888268
rps3-1	12	12	2257	1396	0.7546	0.4225	1.786035503
rps3-2	12	12	1431.5	1398.5	0.7518	0.432	1.740277778
rpl22	11	12	1656	1340.5	0.945	0.4425	2.13559322
rps19	12	12	3028.5	1539	0.9317	0.426	2.187089202
rps19-rpl2ex2	12	12	2545.5	2155	0.8204	0.3595	2.282058414
rpl2ex2-in	12	12	2578	2114	0.7406	0.363	2.040220386
rpl2ex2-in-ex1	12	12	2854	2053	0.7077	0.369	1.917886179
rpl2in-ex1	12	12	2105	1533	0.7028	0.362	1.941436464
rpl2ex1	12	12	2838	2029.5	0.7238	0.329	2.2
rpl23-I-CAU	12	12	2486.5	1689.5	0.8267	0.41	2.016341463
I-CAU-ycf2	12	12	2637	1642.5	0.4186	0.251	1.667729084
ycf2-1	12	12	2424.5	1668	0.4998	0.344	1.452906977
ycf2-3	12	12	2527	1768.5	0.5677	0.3765	1.507835325
ycf2-4	12	12	2806.5	1767	0.5663	0.3625	1.562206897
ycf2-5	12	12	1930	1134.5	0.4816	0.3905	1.233290653
ycf2-6	12	12	2054.5	1384	0.4004	0.3195	1.253208138
ycf2-7	12	12	2576	1797	0.4298	0.348	1.235057471
ycf2-8	12	12	2068.5	1238	0.6727	0.385	1.747272727
ycf2-IG	12	12	1945	1308.5	0.5103	0.3805	1.341130092
IG-L-CAA	12	12	3199	1409	0.532	0.4265	1.247362251
L-CAA	12	12	1960	1465.5	0.4032	0.3535	1.140594059
ndhBex2-1	12	12	2714	1510.5	0.5558	0.3375	1.646814815
ndhBex2-2	12	12	1494	912.5	0.4935	0.3175	1.554330709
ndhBex2-in	12	12	1860.5	1010	0.6237	0.384	1.62421875
ndhBex2-in-ex1	12	12	3609	2210.5	0.595	0.392	1.517857143
ndhBin-ex1	12	12	1115.5	818.5	0.5866	0.3515	1.668847795
ndhBex1-1	12	12	2626	1828.5	0.63	0.3615	1.742738589
ndhBex1-2	12	12	2916.5	1806.5	0.6923	0.3245	2.133436055
IG-rps7	12	12	1529	1031.5	0.525	0.3585	1.464435146
rps7-rps12ex3	12	12	2738.5	2349.5	0.4809	0.293	1.641296928
rps12in2-ex2	12	12	2918	1351.5	0.4886	0.338	1.44556213
rps12ex2	12	12	2611.5	1379	0.4627	0.301	1.537209302
rps12in1	12	12	3528	2074.5	0.4893	0.3275	1.494045802
IG rps12-V-GAC-1	12	12	3616.5	1157.5	0.4872	0.288	1.691666667
IG rps12-V-GAC-2	12	12	1473.5	856.5	0.4081	0.305	1.338032787
IG rps12-V-GAC-3	11	12	1989	1373	0.3892	0.2655	1.465913371
IG rps12-V-GAC-4	11	11	1855	1190	0.3808	0.306	1.244444444
IG rps12-V-GAC-5	12	12	1903	1078.5	0.3276	0.252	1.3
V-GAC	12	12	4210	1512.5	0.3486	0.2175	1.602758621
V-GAC-16S	12	12	20754	15002	0.0245	0.0355	0.690140845
16S-1	12	12	37694	19897	0.035	0.0495	0.707070707
16S-2	12	12	13777.5	7170.5	0.0336	0.057	0.589473684
16S-3	12	12	12399.5	8502.5	0.0476	0.066	0.721212121

16S-4	12	12	11934	7914	0.0385	0.0555	0.693693694
16S-5	12	12	3765	2901.5	0.0987	0.0565	1.746902655
I-GAU ex1	12	12	4515.5	2747	0.1505	0.0795	1.893081761
I-GAU ex1-in	12	12	1199.5	759	0.4165	0.1675	2.486567164
I-GAU ex2	10	12	4034.5	2970.5	0.1708	0.0825	2.07030303
I-GAU-A-UGC	12	12	1172.5	793	0.1848	0.1305	1.416091954
A-UGC ex1-in	12	12	1342.5	589	0.3234	0.184	1.757608696
A-UGC -in	12	12	2122	1153	0.2779	0.1705	1.629912023
A-UGC ex1	12	12	2048.5	843	0.21	0.185	1.135135135
A-UGC-23S	12	12	15389.5	11385.5	0.0245	0.0395	0.620253165
23S-1	12	12	11735.5	7756.5	0.0203	0.0315	0.644444444
23S-2	12	12	3026.5	2855	0.0287	0.048	0.597916667
23S-3	12	12	15407	11179	0.0252	0.04	0.63
23S-4	12	12	22195.5	18027	0.0287	0.0455	0.630769231
23S-5	12	12	12708	10437	0.0252	0.0325	0.775384615
23S-6	12	12	4119	2794	0.0476	0.055	0.865454545
23S-7	12	12	17286.5	15468	0.0329	0.0555	0.592792793
23S-8	12	12	6883.5	6379.5	0.0301	0.0525	0.573333333
23S-9	12	12	8054	7717	0.0252	0.0265	0.950943396
4,5S	12	12	6573	5012.5	0.0553	0.044	1.256818182
R-ACG-1	12	12	3773.5	2478	0.161	0.1335	1.205992509
R-ACG-2	12	12	1954	1027	0.5187	0.4145	1.251387214
N-GUU-1	12	12	701.5	553.5	0.4032	0.277	1.455595668
IG-ycf1	12	12	2871	1672.5	0.4326	0.267	1.620224719
3'ndhF(ycf1)	12	12	2273.5	1209.5	0.8162	0.476	1.714705882
ndhF-1	12	12	2472.5	1336.5	0.9947	0.466	2.134549356
ndhF-2	12	12	2763	1915.5	0.7959	0.458	1.737772926
ndhF-3	12	12	1828	1446	0.7749	0.3935	1.969250318
ndhF-4	12	12	2092.5	1689	0.9625	0.514	1.872568093
ndhF-5	12	12	2118	1142	0.7518	0.4025	1.867826087
ndhF-IG	12	12	385	445	1.0346	0.4165	2.484033613
rpl32	12	12	1121.5	429.5	0.4683	0.188	2.490957447
IG rpl32-ccsA	12	12	740.5	358.5	0.8421	0.5085	1.656047198
IG-ccsA	12	12	596	363.5	0.6069	0.414	1.465942029
ccsA	12	12	2439	3015	0.819	0.3615	2.265560166
ccsA-ndhD	12	12	1055.5	698	0.8722	0.527	1.655028463
ndhD-1	12	12	1038.5	789	0.7238	0.4365	1.658190149
ndhD-2	12	12	764	440.5	0.819	0.4585	1.786259542
ndhD-psaC	12	12	1946	1488.5	1.0458	0.4395	2.379522184
psaC-ndhE	12	12	4656.5	3648	0.6139	0.257	2.388715953
ndhE	12	12	1292	728	0.847	0.454	1.865638767
IG-ndhG	12	12	2647.5	1725.5	0.7917	0.401	1.974314214
ndhG	12	12	1738	1141	0.6909	0.3065	2.254159869
ndhG-ndhI	12	12	2843.5	2681	0.9065	0.418	2.168660287
ndhI	12	12	1164.5	773.5	0.7819	0.418	1.870574163
ndhI-ndhAex2	12	12	826	863	1.0003	0.4835	2.068872802
ndhAex2-in	12	12	862	685	0.7217	0.4715	1.530646872
ndhAex2-in-ex1	12	12	1519.5	1302	0.5964	0.3975	1.500377358

ndhAin-1	12	12	1394.5	878	0.8862	0.5585	1.586750224
ndhAin-2	12	12	786.5	467	0.7784	0.5335	1.459044049
ndhAin-ex1	12	12	962	690	0.7119	0.4805	1.481581686
ndhAex1-ndhH	12	12	1754.5	1411	1.064	0.515	2.066019417
ndhH-1	12	12	957.5	665.5	0.7063	0.4595	1.53710555
ndhH-2	12	12	617	402.5	0.9653	0.5915	1.631952663
rps15	12	12	1145.5	774	0.9037	0.5265	1.71642925
rps15-IG	12	12	902	667.5	0.9212	0.5125	1.797463415
ycf1-1	12	12	1188.5	723	0.9583	0.516	1.857170543
ycf1-2	12	12	1332.5	837	0.9079	0.5275	1.721137441
ycf1-3	12	12	2080.5	1248.5	1.2474	0.645	1.933953488
ycf1-4	12	12	1535	954	0.9744	0.559	1.743112701
ycf1-5	12	12	1545.5	791	1.0619	0.632	1.680221519
ycf1-6	12	12	1714.5	1005.5	0.9975	0.575	1.734782609
ycf1-7	12	12	1457	1026.5	0.7182	0.4825	1.488497409
ycf1-8	12	12	1543	1147.5	0.6475	0.446	1.451793722
ycf1-9	12	12	2504.5	1031	0.6524	0.4605	1.416720955
N-GUU-2	12	12	3482	2457	0.2989	0.1755	1.703133903
IG N-R	12	12	1909	1423	0.6146	0.4665	1.317470525
ycf2-9	12	12	1985.5	1307	0.5684	0.3715	1.530013459
ycf2-I-CAU	12	12	3249	1369	0.3997	0.265	1.508301887
18S	12	12	2193	2103	0.0574	0.061	0.940983607

[1] each probe is spotted with 12 replications per chip; spots with less than 5 detectable spots were removed from the data set

[2] differential enrichment of IP over control- IP, based on the median of ratios of red (635 nm) or green (532 nm) fluorescence
